# A phylogeny for the pomatiopsidae (Gastropoda: Rissooidea): a resource for taxonomic, parasitological and biodiversity studies

**DOI:** 10.1186/1471-2148-14-29

**Published:** 2014-02-18

**Authors:** Liang Liu, Guan-Nan Huo, Hong-Bin He, Benjiang Zhou, Stephen W Attwood

**Affiliations:** 1 State Key Laboratory of Biotherapy, West China Hospital, West China Medical School, Sichuan University, 1 KeYuan 4 Lu, Chengdu, Sichuan 610041, People’s Republic of China; 2 Hunan Institute of Parasitic Diseases, Yueyang, Hunan, People’s Republic of China; 3 Haiyuan College, Kunming Medical University, Kunming, Yunnan, People’s Republic of China

**Keywords:** Pomatiopsidae, Triculinae, Neotricula, Schistosomiasis, Phylogeny, Phylogeography, Taxonomy, Amnicolidae, Biodiversity

## Abstract

**Background:**

The Pomatiopsidae are reported from northern India into southern China and Southeast Asia, with two sub-families, the Pomatiopsinae (which include freshwater, amphibious, terrestrial and marine species) and the freshwater Triculinae. Both include species acting as intermediate host for species of the blood-fluke *Schistosoma* which cause a public health problem in East Asia. Also, with around 120 species, triculine biodiversity exceeds that of any other endemic freshwater molluscan fauna. Nevertheless, the origins of the Pomatiopsidae, the factors driving such a diverse radiation and aspects of their co-evolution with *Schistosoma* are not fully understood. Many taxonomic questions remain; there are problems identifying medically relevant species. The predicted range is mostly unsurveyed and the true biodiversity of the family is underestimated. Consequently, the aim of the study was to collect DNA-sequence data for as many pomatiopsid taxa as possible, as a first step in providing a resource for identification of epidemiologically significant species (by non-malacologists), for use in resolving taxonomic confusion and for testing phylogeographical hypotheses.

**Results:**

The evolutionary radiation of the Triculinae was shown to have been rapid and mostly post late Miocene. Molecular dating indicated that the radiation of these snails was driven first by the uplift of the Himalaya and onset of a monsoon system, and then by late-Pliocene global warming. The status of *Erhaia* as Anmicolidae is supported. The genera *Tricula* and *Neotricula* are shown to be non-monophyletic and the tribe Jullieniini may be polyphyletic (based on convergent characters). Triculinae from northern Vietnam could be derived from *Gammatricula* of Fujian/Yunnan, China.

**Conclusions:**

The molecular dates and phylogenetic estimates in this study are consistent with an Australasian origin for the Pomatiopsidae and an East to West radiation via Oligocene Borneo-Philippines island hopping to Japan and then China (Triculinae arising mid-Miocene in Southeast China), and less so with a triculine origin in Tibet. The lack of monophyly in the medically important genera and indications of taxonomic inaccuracies, call for further work to identify epidemiologically significant taxa (e.g., *Halewisia* may be potential hosts for *Schistosoma mekongi*) and highlight the need for surveys to determine the true biodiversity of the Triculinae.

## Background

### The importance of the pomatiopsidae

**Figure 1:**
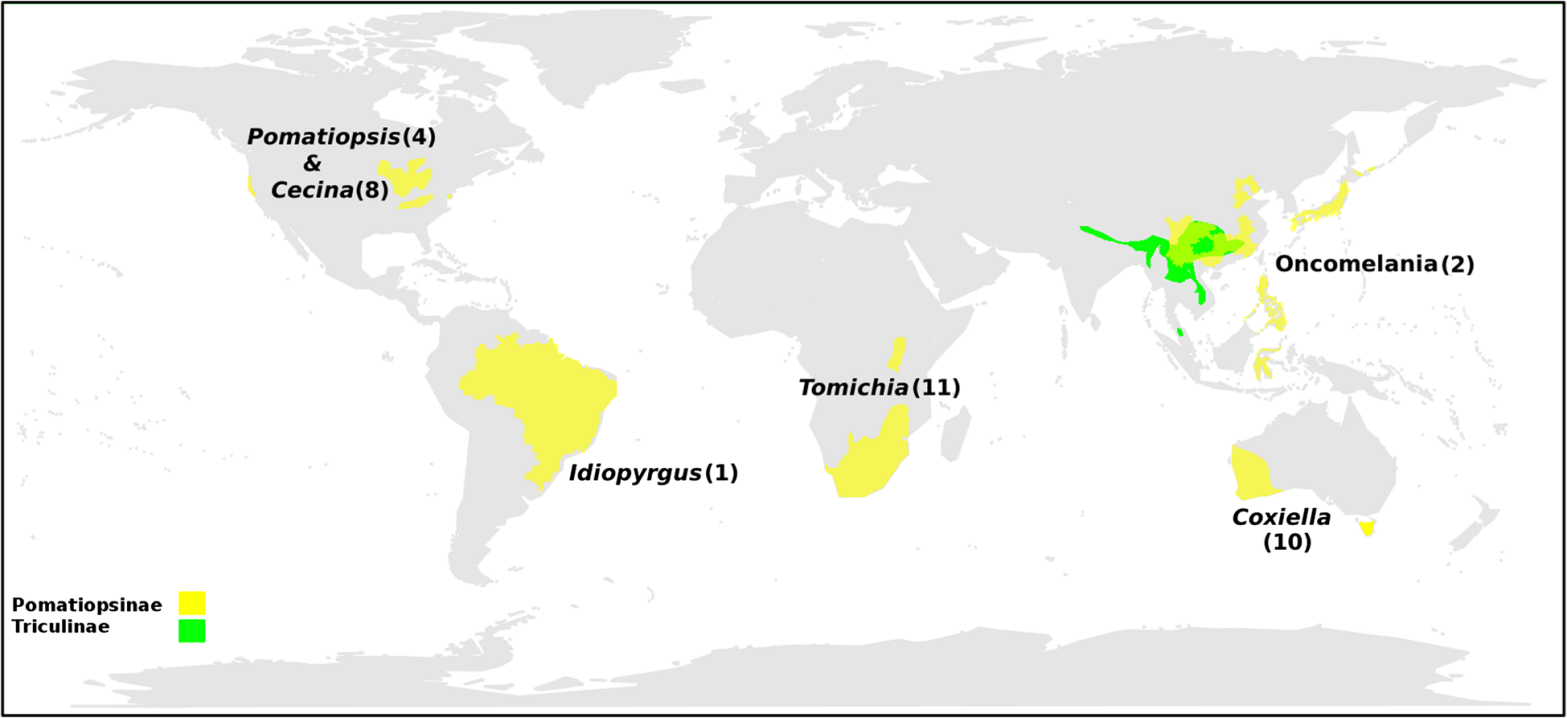
**The current geographical distribution of the Pomatiopsidae.** The known distribution of the sub-families Pomatiopsinae and Triculinae are shown as shaded areas and the locations of key genera indicated, with number of currently recognized species in parentheses. *Coxiella* includes one species recently extinct. *Cecina* is also found in Japan. The range of the Triculinae was plotted using the present data and notes in the literature. The range of *Oncomelania hupensis* was taken from the literature [5]. The distributions of the African and North American taxa are given only to State level. Map generated using the R packages maps and mapdata [6,7].

The Pomatiopsidae comprises two subfamilies, the Pomatiopsinae, with an apparent Gondwanan distribution, and the Triculinae, which are found from northern India into southern China and Southeast Asia (Figure 1). The Pomatiopsinae include *Oncomelania hupensis* subspecies that transmit the parasitic blood-fluke *Schistosoma japonicum* (Trematoda: Digenea) in China, the Philippines and Sulawesi [1]. The Pomatiopsinae are amphibious to terrestrial and freshwater to brackish or marine, which is probably the reason for their much wider distribution. The Triculinae are freshwater and show poor dispersal capabilities, with many species apparently endemic to a single stream, valley or river system. The Triculinae include *Neotricula aperta*, the intermediate host of *Schistosoma mekongi*, which primarily infects humans, mostly along the Mekong River of the Lao People’s Democratic Republic (Lao PDR or Laos) and Cambodia. The total number of people already infected by these two parasites is estimated to be over 50 million [2]. A further five species of Triculinae have been shown to transmit schistosomiasis to humans and/or animals in mainland Southeast Asia [3]. Pomatiopsid snails also act as first intermediate host for the lung-fluke *Paragonimus* and other trematodes [4].

In addition to their parasitological importance, the Triculinae are of great interest owing to their biodiversity, conservation status, and the extent of their adaptive radiation. Radiations of Triculinae occur in Yunnan/Sichuan (Southwest China), 11 species; Hunan (Southeast China), 13 species (mainly *Neotricula*) [8,9]; and an endemic fauna that includes over 90 species occurs along a 300 km stretch of the lower Mekong river in Thailand and Laos [10]. The biodiversity of the Triculinae exceeds that of any other endemic freshwater molluscan fauna. If the endemic gastropods are counted for the ancient lakes, Baikal, Biwa, Ohrid, Tanganyika, and Titicaca, the species of the Mekong river Triculinae outnumber the entire endemic fauna in any one lake and show more than double the number of species found in any one endemic family in a lake. Gastropod radiations in lotic systems are generally more speciose than those in lakes and the Alabama River system (Alabama, Cahaba, Coosa, and Mobile rivers) radiation of the Southeast USA is considered to be a remarkable radiation of endemic freshwater molluscs [11]; however, the lower Mekong river triculine radiation exceeds even this [10]. In addition, the low vagility, high levels of endemism, strict habitat specialisation and sensitivity to environmental disturbance make triculine snails good indicators of near-pristine or long-term undisturbed habitats. All Pomatiopsidae are calciphilic and many are associated with endangered karst habitats; this further enhances their conservation significance.

In spite of the importance of this group many questions remain regarding their biology. For example, the origins of the Pomatiopsidae, the factors driving such a diverse radiation and aspects of their co-evolution with *Schistosoma* are not fully understood. Many taxonomic questions remain and there are still problems assigning known taxa to genera, further, with much of their predicted range unsurveyed (most triculines are highland species), the true biodiversity of the family is certainly greatly underestimated. Public health surveillance is inhibited by confusion between snail intermediate hosts of trematodes and similar looking sympatric species; there is also confusion as to which snails are acting as hosts for *Schistosoma.* The problems of small size (most Triculinae are < 3 mm in shell height), for many species an inability to breed in the laboratory, remoteness and inaccessibility of habitats, a high prevalence of convergent evolution and a relatively high degree of intraspecific variation among anatomical characters, all hamper studies of the Triculinae.

### Taxonomic problems

Questions remain to be addressed at all taxonomic levels concerning the Triculinae. The currently accepted taxonomy is based on that of Brandt [12] which has been extensively revised by G.M. Davis (e.g., [13]). The Triculinae were originally divided into three tribes based on a cladistic analysis which used morphological characters (namely, the Jullieniini, Lacunopsini and Triculini) [10]. In 1990 these clades were revised, a new tribe, Pachydrobiini was defined, thus dividing the Triculini sensu Davis 1979 into the Triculini (*Tricula, Delavaya, Fenouilia, Lacunopsis*) and the Pachydrobiini (*Gammatricula, Guoia*, *Halewisia, Jinghongia, Neotricula, Pachydrobia, Robertsiella, Wuconchona*) [14]. The Pomatiopsinae were also revised following the discovery of a new genus, *Erhaia*, in Yunnan, which was provisionally assigned to a new tribe of the Pomatiopsidae: Pomatiopsinae, the Erhaiini. Genera originally placed in the Pomatiopsinae (*Pomatiopsis*, *Oncomelania*, *Cecina*, *et al.*) were placed in a new tribe, the Pomatiopsini [15]. Further revisions were necessary in 1992, with the creation of a new tribe, the Pseudobythinellini, to accommodate the genera *Akiyoshia* and *Pseudobythinella* which had been recognized as Pomatiopsinae; these included some taxa originally referred to the Erhaiini and so *Erhaia* was placed in synonymy with *Pseudobythinella*[8]. More recently *Erhaia* (and other Pseudobythinellini such as *Akiyoshia*) were transferred to the Amnicolidae: Amnicolinae [16,17]. Similar taxonomic problems concern the genus *Manningiella*, with most (maybe all) of its species re-assigned to the genera *Halewisia* and *Hubendickia*[10]; the fact that these genera belong to different tribes further illustrates the problems encountered in triculine taxonomy. The revision of the Triculinae is a work in progress.

The current systematics of the Pomatiopsidae is largely based on morphological characters. Clearly this is a situation where DNA-sequence based phylogenies would be useful in resolving taxonomic questions. Several authors have published such phylogenies involving Pomatiopsidae, but the focus has been on relationships within medically important genera [3-5,18-21] or the study was addressing questions on a different family [22] or higher divisions in the Rissooidea [23]. A few DNA based phylogenies have been published addressing questions at higher taxonomic levels, but each has been generally restricted to single radiation. For example, a DNA based phylogeny has been published for Chinese Triculinae [24], but this study omitted the Mekong radiation and included only one pomatiopsine snail. The study was also adopted a disputed taxonomy (that of [25-27], which is based solely on characters known to be unreliable such as those of shell, radula and head-foot; the present study uses the better documented taxonomy of Davis [10] and later works by that author. The taxonomy of Davis is based on descriptions of taxa involving more reliable characters and more complete evidence (e.g., more extensive photomicrography, SEM and fine-scale dissection work). The study of [24] found two main clades of Triculinae, with the three taxa they sampled from Fujian, Guangxi and Zhejiang provinces in one clade (together with *N. aperta*), and those from Hunan, Sichuan and Hubei provinces in the other clade; this the authors related to the greater distance of Fujian, Guangxi and Zhejiang from the Yangtze river. These authors also found *Tricula* to be paraphyletic; however, the taxa in question were *T. wumingensis* and *T. fujianensis*, which are taxa described by Liu et al. [25] and Hu et al. [27] based on superficial characters only. The phylogeny published by Kameda and Kato [28] covers a broader range of taxa than that of the 2008 study and is based on both nuclear (18S and 28S rDNA) and mitochondrial (16S rDNA and cox1) DNA sequences; however, it is focused on the Pomatiopsinae and includes Triculinae sequences from GenBank for comparative purposes (these authors followed the triculine taxonomy of Liu et al. [25]; Tang et al. [26]; Hu et al., [27]. The 2011 study provided new sequence data for Japanese *Akiyoshia* confirming that it is not Pomatiopsidae. Finally, there are reports, based on morphology, of Pachydrobiini from Vietnam that are described as *Vietricula*[29,30]; however, these snails resemble *Pachydrobia* and not any kind of *Tricula*.

### Questions regarding the origin and evolution of the pomatiopsidae

#### Tibet (west–east) radiation hypotheses

**Figure 2:**
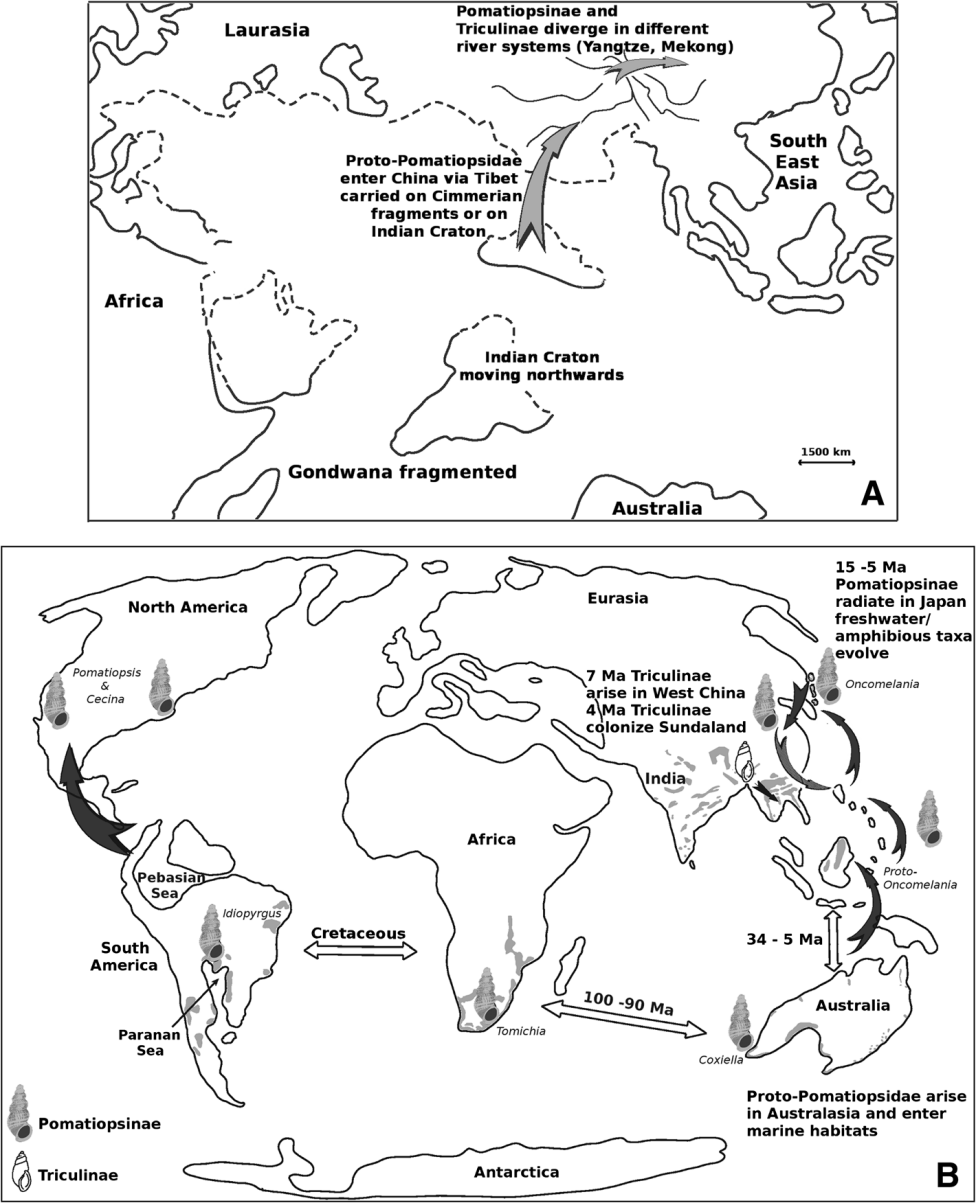
**Summary of alternative phylogeographies for the Pomatiopsidae.** Semi-schematic showing alternative hypotheses for the origins and evolutionary radiation of the Pomatiopsidae. **A**. Tibet hypothesis: The ancestor of the Pomatiopsidae arises in Gondwana and is rafted on the Indian Craton after the break up of the super continent. These taxa are then introduced to China via Tibet after the collision between India (or earlier Cimmerian blocks) and Asia (150–120 Ma). The Pomatiopsinae and Triculinae then diverge in the Yangtze and Mekong river systems as these cut their way southwards to the sea [10]. **B**. Hunan (East to West) hypothesis, as proposed by Attwood (2009) [9]: Proto-Pomatiopsinae diverge in Australasia, with marine forms developing and colonizing South Africa and South America. Precursors of *Oncomelania* colonize northwards along island chains created by low sea levels and by tectonic movements (rafting). After reaching Japan, Proto-*Oncomelania* gives rise to the Japanese Pomtiopsinae and *Oncomelania hupensis*; the latter colonizes China and back-tracks (grey stippled arrow) to recolonize the Philippines and Sulawesi (replacing antecedent forms). The Triculinae arise in Southwest China and diverge in uplifting mountain areas. Dates are from the present analyses. Approximate distributions of major formations of calcareous rocks are shown as shaded areas. Coastlines are rough approximations for 15–10 Ma, drawing from the palaeogeographical literature [31-39].

**Figure 3:**
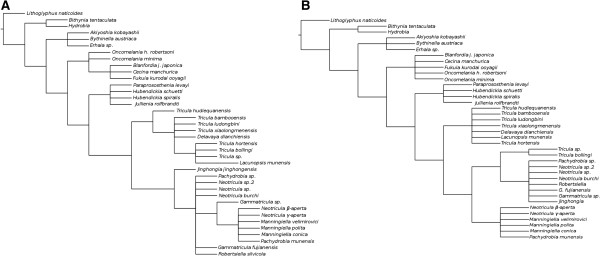
**Phylogenetic trees consistent with alternative phylogeographies for the Pomatiopsidae. A**. Phylogeny consistent with the Tibet hypothesis. Taxa are generally considered to be more derived (i.e., to show progressively more apomorphic character states) as one moves away from Dali in Yunnan, following suggestions in the literature [10]. Taxa are grouped into clades according to hypothesized tracts of dispersal and isolating geographical barriers. **B**. Phylogeny consistent with the Hunan hypothesis (following Table 1).

Differing hypotheses have been put forward to explain the origins, radiation and current biodiversity and deployment of the Pomatiopsidae (Figure 2). Davis [10] proposed a Gondwanan origin for the Pomatiopsidae, with rafting to mainland Asia (via the Indian Craton after the break up of Gondwana) and colonization of Southeast Asia and China (Tibet/Yunnan) via the northern India-Myanmar, Brahmaputra-Irrawaddy river corridor during the mid to late Miocene. The drivingforce behind the radiation of the Triculinae was attributed to the opening up of new habitats along the extending courses of the main rivers of Asia, following their inception at the collision of India and Asia, and the uplift of the Tibetan Plateau. The origin of the Triculinae was located in the highlands of Tibet and Yunnan and dated at around 18 Ma (megaannum or million years). The Gondwana-Indian origin would predict Pomatiopsidae in India and Myanmar/Bangladesh; however, no pomatiopsids have been verified in that region. Species cited from the region, such as *Tricula horae, T. martini, T. taylori* (Myanmar), and even the type species of the genus itself, *Tricula montana* (India) are known only from poorly described, single collections, made decades ago, the identification of which has long been controversial [40]. Indeed many of these taxa more resemble *Oncomelania* than *Tricula,* but it is the distribution of the Triculinae which is key because triculine snails show much poorer dispersal capabilities. Recent reports of *Tricula* from Nepal (e.g., *Tricula godawariensis* Nesemann & Sharma 2007) [41] are simlarly data deficient, with descriptions of new taxa based on shell characters only – as noted by Davis [42], one cannot distinguish these taxa on the basis of shell and radula characters alone. Under this hypothesis the Pomatiopsinae diverge in the Yangtze river drainage and the Triculinae into the Mekong river drainage; this major separation dated mid-Miocene (18 Ma). *Oncomelania* was assumed to have colonized Japan and the Philippines from China, with the radiation into several species of terrestrial Pomatiopsinae in Japan being driven by Miocene tectonic upheaval [10]. The lack of divergence among the other Pomatiopsinae (e.g., *Tomichia* with limited geographical or habitat range and few species having evolved over 80 Ma; see Figure 1) is explained by environmental stability [43]. Nevertheless, such a lack of radiation in southern continentally distributed pomatiopsines over 80 Ma compared with the extensive radiation of the Japanese taxa over 23–5 Ma is not easy to accept, and also the lack of radiation on the tectonically active Philippines (only one sub species of *O. hupensis* is found there) requires explanation. The phylogeny, dispersal tracts and time markers associated with this hypotheses are summarized in Table 1 and Figures 2 and 3A.

**Table 1:** Phylogeographical events and divergence time predictions used to construct phylogenies corresponding to, and in accordance with, alternative hypotheses for the origin and radiation of the Triculinae and related taxa

**Hypothesis … Event**	**Location/rationale**	**Predicted date [source]**
*Tibet (West–east) radiation*[10]		
1. Lithoglyphidae diverge from Hydrobiidae	Pangaea/fossil record [44]	305-169 Ma [44]
2. Amnicolidae and proto-Pomatiopsidae diverge	Pangaea after initial breakup into northern and Gondwanaland continents/amnicolids common and diverse in North, but less so in Asia, therefore it is assumed that they crossed into Southwest China before the major Himalayan uplift) [10]	200-190 Ma [45]
3. Pomatiopsidae arise after break up of East Gondwana	Gondwana/Fossil record indicates no Triassic rafting of pomatiopsids on West Burma block and Cimmeria to Asia	<210-150 Ma [46]
4. Pomatiopsinae and Triculinae diverge	Indian Craton - after separation from Gondwana (as no Triculinae in South Africa) [10]	≤165 Ma [46,47]
5. The tribes of the Triculinae diverge	Yunnan/Prior to closure of the Brahmaputra-Irrawaddy-Mekong corridor (because all three tribes of the Triculinae are found in lower Mekong) [10]	*c.a.* 18 Ma [10]
6. *Lacunopsis* clade diverged from Triculini	Yunnan/*Fenouilia* of Yunnan, like *Lacunopsis,* is derived from proto-*Tricula bollingi*[48] therefore, date lies between divergence of Triculini (18 Ma) and major regional uplift	18-7 Ma [49]
7. Lower Mekong triculine radiation in Laos	Triculine taxa become more derived as they radiate out from Dali/evolution occurs in concert with the evolution of the rivers as they cut southwards towards the sea [10]	11-5 Ma [31,50]
*Hunan (East to West) radiation*[9]		
1. Lithoglyphidae diverge from Hydrobiidae	Pangaea/fossil record [44]	305-169 Ma [44]
2. Amnicolidae and proto-Pomatiopsidae diverge	East Gondwana/After separation from India-Madagascar (because no confirmed reports of pomatiopsids in India, either extant or fossil)	*c.a.* 132 Ma [46,47]
3. Pomatiopsinae diverge in marine habitats	Eastern Gondwana (Australia)/inundation by high sea levels, increased coastal habitats	*c.a.* 112 Ma [51]
4. Proto-*Oncomelania* arises	Tertiary island hopping along extensive island complex (Borneo-Philippines) [9]	40-20 Ma [47]
5. Radiation of terrestrial/amphibious pomatiopsines	Japan/Miocene orogeny in Japan plus local and global climate change [9]	15-5 Ma [39,52]
6. *Oncomelania* enters China and diverges	On Yangtze plain/Prior to opening of Sea of Japan [53]	*c.a.* 15 Ma [54]
7. Triculinae diverge from proto-*Oncomelania*	West China/Triculinae adapt to new conditions from Pliocene major uplift of Himalaya [53]	*c.a.* 7 Ma [49]
8. Hunan, northern Lao & Vietnam Pachydrobiini isolate	Indosinia/Red river corridor between southern China (Hunan) and Sundaland broken [20]	*c.a.* 4.4 Ma [55]
9. Yunnan & Sichuan Triculinae isolate in uplifting terrane	Southwest China/Uplift of Hengduan mountains and associated ranges	*c.a.* 3.4 Ma [56]
10. *Lacunopsis* clade diverged from Triculini	Yunnan/Prior to Hengduan orogeny	*c.a.* 3.4 Ma [56]
11. Northern Thailand Triculini isolated from China/Sundaland taxa	Late-Pliocene block faulting causes several course changes along the proto-Mekong river [2] North Sundaland/Extended Mekong-Ping river separation and flow reversal [20]	*c.a.* 3.4 Ma [56,57]
12. Lower Mekong extensive radiation of Pachydrobbiini and other Triculinae	Climatic change and range contraction/fragmentation	*c.a.* 2.5 Ma [58]
13. *Robertsiella* diverges from Mekong Pachydrobiini in Pahang river drainage, Malaysia	West Malaysia/Mekong river course changes and rising sea levels flood Sunda shelf, break river connections between Cambodia & Malaysia [59]/divergence of *S. malayensis*[60]	2.6-0.8 Ma [61]
14. Divergence of Erhai & Dianchi Basin taxa in Yunnan	Yunnan/Stage 3–2 of Sanjiang orogeny in Yunnan and associated tectonic events	0.9 Ma [49]
15. *Neotricula aperta* strains diverge	Final surge of Himalayan uplift, tilting of Khorat Basin, Mul-river flow reversal, and volcanism in southern Laos [19], drive further divergence in the lower Mekong	0.8-0.9 Ma [49]

Citations accompanying dates represent sources of dates for palaeogeographical or tectonic events; citations in the rationale column refer to sources of explanations given; those entries in the rationale column without citations refer to explanations being put forward *de novo* in the present study.

Very little fossil evidence has been presented in the literature in support of the India rafting hypothesis and Miocene entry of Pomatiopsidae into eastern Asia, from India, via Tibet. Fossils resembling *Tomichia* have been reported from the upper Cretaceous of South Africa [62]; however, this is after the currently accepted date for the separation of Africa and India, and a fossil (being shell only) provides insufficient characters for the identification of hydrobiid genera. More recently Neubauer and colleagues [63] reported what they described as a triculine genus from the upper most Eocene to lower Oligocene of nothern Vietnam, namely *Bacbotricula* gen. nov. (Pomatiopsidae: Triculinae). The fossil was assigned to the Triculinae because, despite the name *Bacbotricula,* it resembled jullieniinine taxa, such as *Hydrorissoia, Karelainia, Neoprososthenia* and *Paraprososthenia,* in having a turreted shell with spiral sculpture; however, no extant Triculinae shows such a marked keel and angulated parietal wall as “*Bacbotricula*”. On the basis of the shell alone, this taxon could be Rissooidea or small Cerithioidea, and the authors did note that their taxonomy was tentative. In the present study fossil evidence is used to date only the deepest cladogenic events, and such data are not suitable for application at higher taxonomic levels. Further, if we date the find as Priabonian-Rupelian (40–28 Ma), *Bacbotricula*’s arrival in Vietnam appears to pre-date the evolution of the rivers of Yunnan, which are proposed to have driven and shaped phylogenesis among the Pomatiopsidae under the Tibet hypothesis. Nevertheless, Neubauer et al., have provided data consistent with the idea that there could have been Triculinae in Oligocene Vietnam and an insight into their freshwater ecology; these authors also proposed the Red river as a possible route for triculine snails to have entered Southeast Asia from Tibet/Yunnan, thus echoing the “Red river hypothesis” [2] of earlier papers (see following sub-section).

#### Hunan (east to west) radiation hypothesis

As mentioned above the Tibet hypothesis fails to explain the current deployment of triculine taxa. For example, the lack of *Tricula spp.* (and *Oncomelania*) in India, the absence of *Neotricula* from Yunnan and its mono-specific radiation in Southeast Asia. Estimated dates of divergence for Asian *Schistosoma spp.* also conflict with the Tibet hypothesis. DNA sequence data supported a 3–6 Ma age for the *Schistosoma sinensium* group (the clade to which *S. malayensis, S. mekongi,* and *S. japonicum* belong [64]), with the divergence of *S. japonicum* at around 4 Ma and *S. malayensis/S. mekongi* at 2.5 Ma [60]. An alternative hypothesis was proposed which explained the deployment of the Triculinae as a mid-Pliocene radiation from Hunan in southern China, westwards into Southeast Asia [9,20]. At least 5 species of *Neotricula* are known from Hunan and the closest relatives of *Robertsiella* (*Guoia* and *Wuconchona*) are found in Hunan and not in Yunnan or Southeast Asia [8]. The apparent “southern continental” distribution of the phylogenetically basal Pomatiopsinae can be as readily explained by correlation with karst or limestone distribution as by a Gondwanan origin [9] (see Figure 2B).

The 900 km long valley of the Red River fault was proposed as the mid-Pliocene dispersal corridor for proto-*Neotricula* through the Mesozoic Annam mountain chain which forms a barrier between Hunan and northern Laos/Vietnam [2]. In the past the Red River fault ran 400 km further West of its current path, and therefore closer to Laos [65]. In addition, the Yangtze river is believed to have flowed along a common course with the Red River [50]. The Red-River radiation into Southeast Asia was dated at *c.a.* 4 Ma, just prior to major tectonic events in the region which may have led to a Pliocene switch from previously southwards courses to the eastwards courses seen in the major Chinese rivers today [57]; this change in the drainage pattern would have been a significant isolating event in the region [60]. The Pliocene Dong-Ngai-Mekong River, which once flowed North to South across the Sunda shelf (now off shore of Vietnam’s East coast) to the East of the Annam mountains [61], could have introduced proto-*Neotricula* to Southeast Asia from Hunan. The ancestral condition of Triculinae is spring or primary stream dwelling, and this is seen in almost all Triculinae. *Neotricula aperta* and other lower Mekong triculines are unusual in that they have become adapted to life in large rivers such as the Mekong. This adaptation may have been a response to climatic change and range contraction/fragmentation occurring 2.5 Ma following a marked intensification of monsoon winds affecting rainfall patterns in the region [58]. Cambodia would have been the most likely point of entry into Southeast Asia from the vast plain of wetlands and rivers that existed off the present day coast of Vietnam, with a similar plain linking Cambodia and West Malaysia. The uplift of volcanic highlands in Southeast Cambodia at 0.8 Ma [66] probably cut off the Vietnam-Cambodia corridor just South of the Kontum range. The Pleistocene Mekong river flowed West, along the present day border between Thailand and Cambodia, then southwards along the Tonle Sap river of today, from where it flowed across the exposed Sunda shelf towards Malaysia [59]. The lower Mekong River is unlikely to have occupied its present course until 5–6 ka (thousand years ago) [31]. Consequently, the Triculinae of the lower Mekong river most likely radiated in different habitats and river systems until the Holocene. Under the East to West hypothesis, the Triculinae are assumed to have diverged from the Pomatiopsinae as these taxa migrated westwards and were caught up in the rapidly uplifting terrain of the Shan and north Indosinian mountains (Yunnan/northern Laos). *Tricula* (and other Triculini) was proposed to have diverged in the higher elevations of Yunnan and *Neotricula* (and other Pachydrobiini) in the small streams of the lesser mountains of Hunan. In this model *Oncomelania* is regarded as diverging from other Pomatiopsidae, which are ancestrally marine or brackish water, in Australasia and colonizing China from Japan and eastern China (Figure 2B), rather than from India via Tibet and Yunnan [9].

The Triculinae are proposed to have arisen from a common ancestor with Pomatiopsinae which arose in the Cretaceous on eastern Gondwana (Australia-Antarctica-Papua New Guinea-New Zealand etc.) after separation of India/Madagascar (because Pomatiopsidae are absent from India and Africa). Rather than rafting via India, the proto-Pomatiopsidae are considered to have rafted from Northwest Australia on small continental fragments of Gondwanan origin which today form parts of Borneo and eastern Indonesia [9]. Onward dispersal to the southern Philippines, then in a more southerly location than at present, would have been via island hopping across the extensive island complex that formed Oligocene North Borneo (Figure 2B). In addition, northwards rafting and rotation brought southerly Gondwanan terranes to abut the Philippines during the Mio-Pliocene. Colonization of Japan would have occurred about mid-Miocene, followed by invasion of the Yangtze plain of China from its East coast. Allozyme and DNA-sequence based phylogenies for *Oncomelania* show the “southern continental” pomatiopsines and Japanese *O. minima* as basal to all other *Oncomelania*[67,68], suggesting that *Oncomelania sensu stricto* arose in Japan, with *O. hupensis* then colonizing China and back-tracking into the Philippines and Sulawesi (probably displacing its ancestral form). The proto-pomatiopsid leaving Gondwana most likely held the ancestral condition and was marine; the dispersal to south Africa and South America (originally in much closer proximity) was therefore not unlikely. Indeed, continental connections or close proximity of southern Africa and South America may have persisted until *c.a.* 105 Ma [69]. The March 2011 Pacific tsunami demonstrated that large aggregates of material may cross the ocean in less than 15 months and that these may harbour viable communities of exotic aquatic organisms (including molluscs) [70]. This hypotheses not only explains why genera such as *Tomichia* show relatively little diversity, but also why the basal genera in the pomatiopsid phylogeny are not those best adapted to mountainous conditions but are those adapted to marine or freshwater wet-land conditions. The phylogeny, dispersal tracts and time markers associated with this hypotheses, together with additional references and details are further explained by Table 1 and Figures 2B and 3B.

### Aims of the study

In view of the many questions regarding the taxonomy of the Triculinae the objective of the present study was to collect samples of as many relevant taxa as possible and to obtain DNA sequence data for representatives of all genera which could be reliably identified by morphological criteria. These data would then be combined with existing published data and phylogenies then estimated by several independent methods so as to obtain a well supported evolutionary history for these taxa. The resulting phylogeny would be used to assess the monophyly of clades within the Triculinae. In addition, alternative hypotheses regarding the origin of the Triculinae were to be tested, and the timing and dispersal tracts of their radiation estimated. The findings would then be used to provide guidance for species identification in the Triculinae, information of use in studies of host range in *Schistosoma* and *Paragonimus* and of general investigations into host-parasite co-evolution, clarification of higher taxonomic groupings in the Pomatiopsidae, and to shed light on the nature and triggers of rapid and highly speciose radiations of freshwater invertebrate taxa. Finally, the work was expected to facilitate conservation and biodiversity studies through clarification of the numbers of taxa present, their habitat and geographical ranges and through aiding identification.

It should be noted that, although the Tibet hypothesis is based on that of Davis [10], the original hypothesis published by Davis was not presented in a form which could be tested relative to a Hunan hypothesis. Consequently, the Tibet hypothesis is an adaptation of the original ideas presented in Davis [10] and other papers. The Hunan hypothesis is also not exclusive of the ideas presented by Davis, for example it looks to Davis [10] for the origin of the Jullieniini and Lacunopsini and also begins with the break up of Gondwana. In view of this, the subject of this paper is not a comparison of a Davis hypothesis with that of other authors, it is more accurately a comparison of two hypotheses, both influenced by the work of Davis, albeit to different degrees.

In this study we estimate phylogenies from DNA-sequence data available at homologous loci for as comprehensive a sample of Pomatiopsidae as possible (16 out of the 32 validated pomatiopsid genera are sampled, including at least 3 taxa which are apparently extinct). A relaxed molecular clock is applied to the phylogenies and key divergence dates estimated by a Bayesian approach. The divergence dates and phylogeny are shown to agree with predictions for the Hunan hypothesis as well, or even better than, those of the traditional Tibet hypothesis. Major cladogenic events are linked to palaeo-climate and geographical changes in the region which could have driven divergence. Phylogenetic affinities are linked to dispersal and divergence along past dispersal corridors. The medically important genera *Neotricula* and *Tricula* were not found to be monophyletic and suggestions are made for their revision.

### The use of vicariant calibrations and panbiogeography

The approaches used in phylogeography have been the subject of much controversy, with objections raised regarding the assumption of vicariance [71], dating using molecular clock rates [72], and the correlation of the age of taxa with the dates of palaeogeographical events [73]. For example, a correlation between continental breakup and phylogeny may only occur where the continental biota was homogeneous prior to the tectonic events [73], and multiple events, occurring at different times, can generate the appearance of false and misleading vicariance (“pseudo-congruence” [74]. The present study has attempted to avoid these problems by adopting a more panbiogeographical approach. The aim is not to use the data to infer or prove dispersal and vicariance events for the phylogeny of the Pomatiopsidae, instead the approach is to compare patterns in the phylogeny with the two main hypotheses for the historical biogeography and evolution of these snails and to assess their relative degrees of fit. Neither of these hypotheses was formulated in the present study, and the events or molecular clock rates used in this study are for the most part independent of those used to derive these hypotheses. The earliest divergences in the hypotheses do assume that cladogenesis is due to plate movement and not post-breakup dispersal or divergence in allopatry prior to isolation by continental fragmentation. Such assumptions have been heavily criticised [71,75]; however, these criticisms often refer to speciation events. In the present study such assumptions are applied mainly to deep cladogenic events and at continental scales, where entire stem lineages fail to enter certain regions (e.g., proto-Pomatiopsidae into India). The events hypothesized are also deep in time (e.g., Cretaceous) so that the gradual nature of tectonic upheavals have less of an effect when treated as events rather than episodes, a potential problem noted by critics [71]. In addition, the more recent dating events in the hypotheses are often seen as triggers of cladogenic eposides (e.g., climate changes after major Himalayan uplift driving triculine radiation) rather than the simple isolating events in vicariance-dispersal approaches. A further criticism of phylogeographical studies is the use of present day regions (e.g. Borneo, China, Southeast Asia) as biogeographical units, and the failure to recognize that these regions can have composite, independent, affinities [73], such as the past connections between parts of Borneo and China or Australia and Indonesia, recognized by the present study, or be influenced by extinct landforms such as the Oligocene island chain north of Australia. Even critics of the use of vicariant calibrations concede that their use can be appropriate in certain cases such as the evolution of freshwater taxa of low vagility [71]. Nevertheless, some assumptions do remain in the present study, such as the assumption that the ecological habit of the Triculinae has only changed once in their history (from marshland to spring or primary stream dwelling), that the freshwater Pomatiopsinae could not cross open seas (whereas their marine ancestors presumably did in the Cretaceous) and that taxa dispersed always and whenever a route became open to them. In view of these considerations, as with all phylogeographical reconstructions, it is necessary to consider the hypotheses presented in this study as possible scenarios rather than definitive histories. Such histories provide frameworks for further enquiry and testing.

The use of molecular clock rates as calibration data, rather than the fossil record, has also been criticised [72,73]. Numerous examples have been cited where the use of molecular clock rates for fishes led to estimated divergence dates either pre-dated the event attributed to their isolation [76-78] or the existence of their ancestral habitat [79,80]. Such criticisms are now rather dated, and all the papers just cited used either a non-parametric method or a penalized likelihood approach (e.g., as used in the software r8S [81]), as was common at the time; the present study uses a Bayesian approach, which not only allows calibration dates to be incorporated as statistical distributions (reflecting the uncertainty of fossil dates or the gradual processes of continental breakup), but also gives more reliable results where a small number of loci are used [82] (see Phylogenetic methods: parameters and model priors). Further, most of the examples of problematic analyses cited in critiques of phylogenetic dating [72,73] are of papers which used either clock rates, or fossils or palaeogeographical events, but not all three sources (as in the present study). Nevertheless, there are important caveats for the use of molecular clock rates. For example, it is highly problematic to use a clock rate from a study which employed a calibration date, e.g., a dated vicariance event, in a subsequent study which aims to date same vicariance event as used in the earlier study [83]. Consequently, in this study care has been taken to choose calibration sources which are as independent as possible from the hypotheses being tested, and to give full details of the priors (calibrations) being used and, where secondary calibrations are used (e.g., clock rates from other studies), of the calibrations used in the primary studies. Further details of calibration points and sources of error in using fossil calibrations are given in Section Hypotheses testing.

## Results

### Sequence data

The PCR primers used in this study amplified a 598 bp stretch of legible DNA sequence at the *cox*1 locus and 541 bp at the *rrn*L locus. The haplotype diversity was 1.000 for the *cox*1 data but only 0.999 for the *rrn*L data (the DNA sequences observed at this locus for *Manningiella conica* and *Manningiella polita* were identical). The combined data set was 1139 bp (1061 bp excluding sites with gaps/missing data) and comprised of 1177 characters including indels (coded by SIC) and alignment gaps/missing data.

Tests for deviations from neutrality were not significant: *cox*1 Fu* Li’s D* -0.37712 (*P* > 0.10), F* -0.73853 (*P* > 0.10); *rrn*L D* -1.46885 (*P* > 0.10), F* -1.82856 (*P* > 0.10), all calculations were based on total number of mutations and the outgroup was specified. Tajima’s D: *cox*1 -1.39619 (*P* > 0.10); *rrn*L −1.63067 (*P* > 0.05). The test of Xia et al. [84] did not indicate significant saturation in the sequence data (Iss 0.295, ISS.cSym 0.752, *P* < 0.0001, a lack of statistical significance here would imply a poor phylogenetic signal). Also, plots of transitions and transversions against distance suggested negligible levels of saturation at both loci.

### Phylogeny reconstruction

#### Maximum likelihood

**Figure 4:**
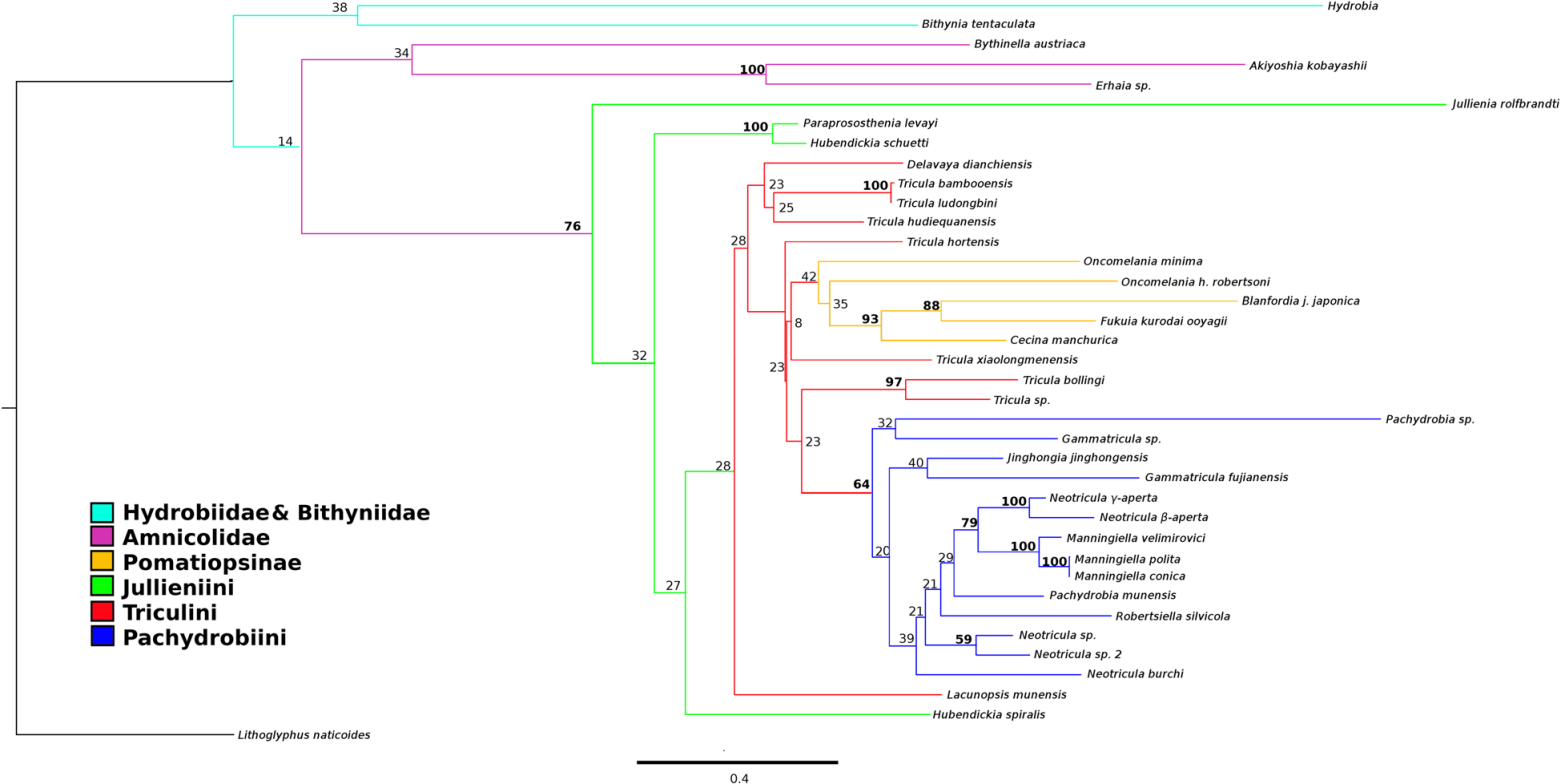
**Best-scoring tree found by ML search with RAxML.** Bootstrap support values are indicated on the tree as a percentage of 100000 replicates. Support values greater than 50% are shown in bold. The outgroup was set as *Lithoglyphus naticoides*. Colour scheme: Lacunopsini are classed with Triculini.

Figure 4 shows the best scoring ML tree estimated using RAxML with all the available data (including coded indels) and *Lithoglyphopsis naticoides* as the outgroup; the log likelihood of this tree is −11216.355271 (note: likelihoods estimated by RAxML under the current settings may differ from those of PAUP* and other similar programmes with the same data). The tree shows the following groups to be monophyletic: Bithyniidae/Hydrobiidae, Amnicolidae, Pomatiopsinae, Pachydrobiini and the genus *Manningiella*. The Jullieniini are found together at the base of the Pomatiopsidae, although polyphyletic. The tree shows several most unexpected relationships: the Triculinae and Triculini are not monophyletic, nor are the genera *Neotricula* and *Tricula. Lacunopsis* is clustered with the Jullieniini (rather than the Triculini) and the Pomatiopsinae arise within the Triculini (rather than at the base of the pomatiopsid clade, distinct from the Triculinae). Bootstrap support on many of the clades is low (note this is not a consensus tree); the only clades with support greater than 50% are the Japanese Pomatiopsinae, the Pachydrobiini, the triculine radiation of the lower Mekong river, and two pairs of sister species.

The ML tree was also estimated for the data with the indels treated as missing data (not coded) (Additional file 1: Figure S1), for comparison with Figure 4. The trees differ in that the Amnicolinae are not monophyletic, *Hubendickia spiralis* is not basal to the other two Lao Julliennini, and the two *Pachydrobia* species are in slightly different locations on the tree. As the clade support values were similarly low and the Amnicolinae were polyphyletic, the inclusion of the indels was considered to be non-detrimental to the estimation of the phylogeny of the taxa in the present study.

#### Bayesian without clock model

The harmonic mean of the marginal likelihoods for the Beast runs was −11231.23; ASDSF was 0.001015 and PSRF values for all parameters were 1.000. Clade credibility values ranged from 32 to 100 percent (only 8 nodes had values < 50%). The topology of the tree representing the phylogeny estimated by MrBayes differed from that estimated by Beast in the following aspects. In the MrBayes tree the Jullieniini are basal at the root of the Pomatiopsidae (not derived from the same ancestor as the West China Triculini) and the Pomatiopsinae are found in the same clade as all Triculinae except for those of West China. In addition the two *Oncomelania* species are not monophyletic in the MrBayes phylogeny and the *Gammatricula* clade is not well resolved. The topology of the MrBayes tree is identical to that of the best-scoring tree found by RAxML. The Beast tree is described below.

#### Bayesian with clock model and date priors

**Figure 5:**
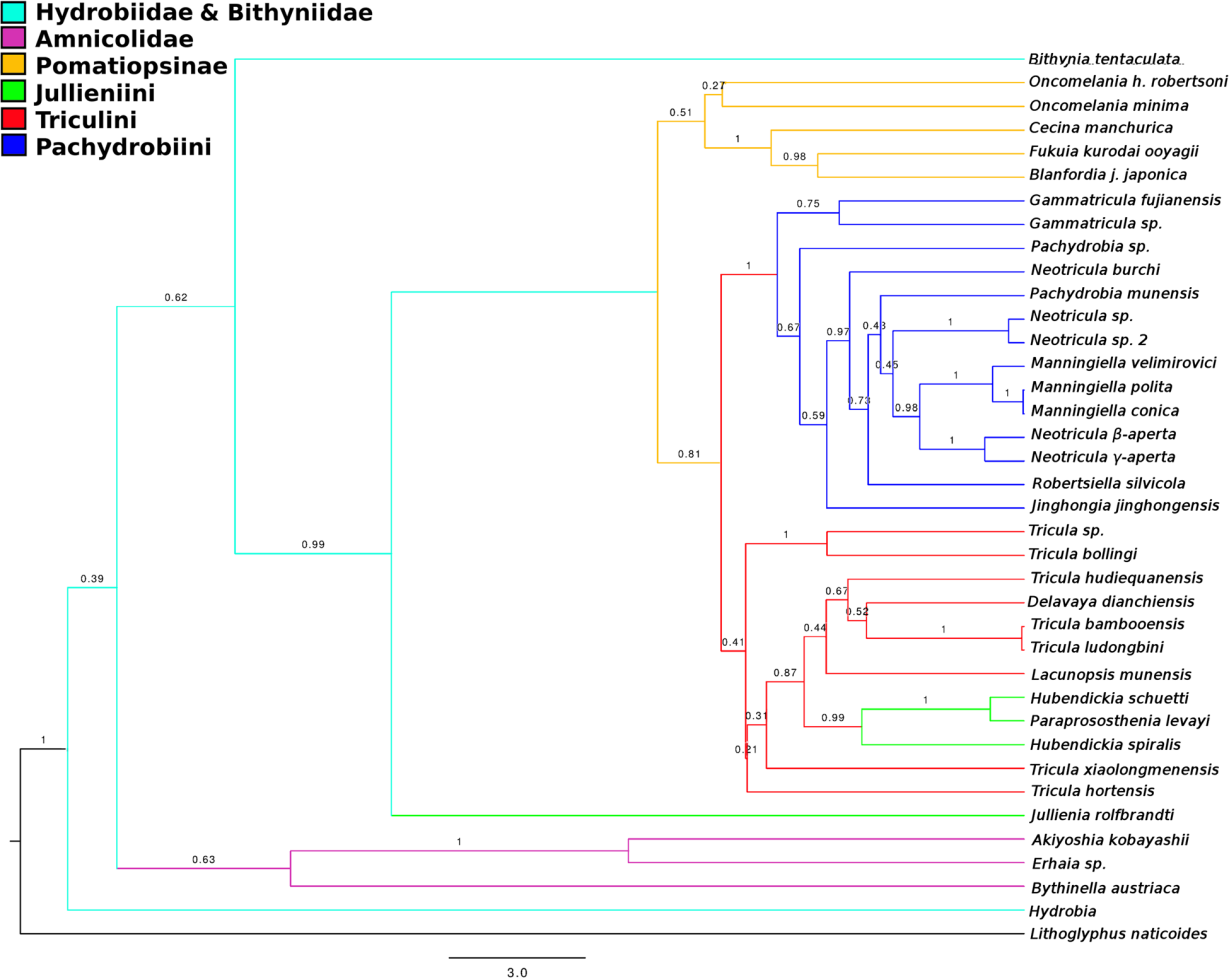
**Maximum clade credibility tree estimated by Beast.** Ultrametric tree estimated using Beast with “Recent” dating priors (see Hypotheses testing in Methods) and 486540000 generations after burnin. Clade credibilities are shown for each node on the tree. No outgroup was specified, but this tree has been rooted at *Lithoglyphus naticoides.* Colour scheme: Lacunopsini are classed with Triculini.

Figure 5 shows the phylogeny estimated by Beast with all three date priors (see Table 2); this is the MCCT (Maximum Clade Credibility Tree) over all 6000 trees sampled post burnin. The posterior probabilities on the clades ranged from 0.21 to 1.0 (78% of nodes showed support values > 0.50). The posterior probability of the tree itself is −10924.1375 ± 0.1375. The relationships within the West China Triculini clade were the least well supported, as was the relationship between the Jullieniini and the Triculini (*P =* 0.21). The Amnicolidae form a fairly well supported monophyletic group (*P* = 0.63); the position of *Hydrobia* is not well supported (*P* = 0.39) and it is located adjacent to the root of the tree, whereas *Bithynia tentaculata* is found at the root of the Pomatiopsidae (with good support *P* = 0.99). The Jullieniinine clade is well supported (*P* = 0.99), but *Hubendickia spiralis* is found at the root of the Pomatiopsidae (after *Bithynia*) and this is well supported (*P* = 1.0). The West China Triculini appear to have arisen at almost the same time as the Jullieniini, and both clades appear to stem from the Triculini of northern Thailand (although the support for this is weaker *P <* 0.35). The pachydrobiinine radiation of Sundaland, together with *Gammatricula* and *Jinghongia,* form a large well supported (*P* = 1.0) clade distinct from the Triculini/Jullieniini clade, which appears to have arisen from Chinese and North Vietnam Pachydrobiini. In addition to the position of the Jullieniini, the Beast tree differs from those of RAxML and MrBayes most strikingly in that it shows the Pomatiopsinae to be a monophyletic clade stemming from the base of the triculine clade (*P* = 0.51). The two species of *Oncomelania* are also seen to form a monophyletic, though poorly supported, clade (*P* = 0.27). Additional file 2: Figure S2 provides a 50% majority rule consensus tree for the 6000 sampled trees and serves to highlight uncertain areas in the phylogeny (i.e., where polytomies occur on the tree).

**Table 2:** Palaeogeographical/fossil record (FR) calibration points used as priors molecular dating

**Prior code/taxa diverging**	**Date**	**Type**	**Geological events involved/biogeography**	**Prior**
Root/Lithoglyphidae diverge from other hydrobioids	305-169	F	Rissooidea in Carboniferous – Hydrobiidae widespread by upper Jurassic [44]	237 ± 34.7
Core group/Proto-Pomatiopsidae and Amnicolidae	161 - 83	F	Pomatiopsids, bithyniids & truncatellids in Purbeckian [85,86] – Hydrobiidae well diverged by Santonian-Campanian [87]	122 ± 19.9
Malaya/*Robertsiella* diverges from Sundaland *Neotricula* clade	2.0 ± 1.98	M	*S. malayensis* and *S. mekongi* diverge (*cox1* and *rrn*S clock calibrated using Pliocene pulse of Tian Shan uplift between C Asia and NW China [88]) [60]	2.0 ± 1.01
Khorat/β and γ strains of *N. aperta* diverge in N E Thailand	c.a. 0.8	P	Volcanism on eastern margin of Khorat Basin and river flow reversal [19]	0.8 ± 0.10

All priors are normally distributed ± Standard Deviation (SD); the SD values reflect the degree of confidence in the calibration point and are set to cover the range of values in column two (95% confidence interval). All times are in Ma. Note: “Core-group” refers to all taxa excluding *Hydrobia* and *Lithoglyphus*. Calibration types: F, fossil record; M, molecular clock; P, palaeogeographical or tectonic.

#### Parsimony method with POY

POY5 was used to implement a parsimony method using dynamic homology characters with direct optimization (i.e., no *a priori* multiple sequence alignment was required). In the initial sensitivity analyses the maximum MRI value (0.97990256) was obtained for a scheme with CP_112_ and *rrn*L partitions and cost weightings 6481; however, this was little different from the simpler scheme of *cox*1 and *rrn*L as one single partition and cost scheme of 6411 (MRI 0.96813765). Consequently, the data were used unpartitioned, Ts and Tv costs were both 1, and gap cost was 64. The MP analysis was found to be much more sensitive to cost weighting than to partitioning scheme.

The estimated phylogeny failed to recover any of the well recognized hydrobioid taxonomic groups (Additional file 3: Figure S3). Neither the Amnicolidae, Pomatiopsidae, Pomatiopsinae, Triculinae nor any of the three tribes of Triculinae appear monophyletic on the tree. The results of the POY analysis failed to shed any light on the evolutionary relationships of these taxa.

### Hypothesis testing

#### Shimodaira-hasegawa test (SH-test) and topological method tests

Constraint trees were drawn to represent the two main hypotheses regarding the evolution of the Triculinae, the Tibet origin with a West to East radiation and the Hunan origin with an East to West radiation (Figure 3). The log likelihood of the best-scoring tree found with a heuristic unconstrained search with PAUP* was −11378.017 and those for the same search constrained by the Tibet hypothesis and the Hunan hypotheses were −11449.67498 and −11447.08823, respectively. These findings can be used in LRTs as a crude comparison of the fit of the hypotheses to the data. In both cases the likelihood under the hypothesis was significantly lower than that of the unconstrained search (*P* < 0.0001); however, there was no significant difference when the likelihoods under the two models were compared with each other (*P* = 0.9999). The SH-test suggested that the constrained tree for the Hunan hypothesis was significantly worse than the unconstrained tree (P = 0.0056), as were 5/14 of the MP trees found by PAUP*. For the Tibet hypotheses, again the constrained tree was significantly worse than the unconstrained tree (P = 0.002409) as were the same five MP trees.

The topology based test (using slightly modified versions of the hypothesis trees, see Hypotheses testing in Methods Section) showed that 0/200100 trees in the posterior distribution were compatible with the Tibet hypothesis (*P* = 0.0000) and 35/200100 with the Hunan hypothesis (*P* = 0.0002). Consequently, all of the tree based hypothesis tests indicate that the Hunan is a slightly better fit to the data, although this difference is not significant and neither hypothesis agrees well with the data (both show a significantly poorer fit than the unconstrained tree).

#### Estimation of divergence times and substitution rates with Beast

The priors used to calibrate the divergence times on the tree and, thereby the substitution rates, are given in Table 2. Two of these (Core-Group and Malaya) were chosen because they are independent of the time markers used to develop the Tibet and Hunan hypotheses (which are given in Table 1). Table 3 provides the mean values of the sampled trace across the McMC chain for substitution rate estimates from Beast for all data partitions (together with SE and 95% HPD). Beast estimates these rates as the total number of substitutions per site divided by the time depth of the tree (in Ma). As expected the rate for the third codon positions was much greater than those for the first or second codons (e.g., 25.54 vs 0.71 and 0.09, respectively (recent divergences runs)) and this relationship was also observed with the P4 and MrBayes analyses and is one indicator that the results have not been affected by a bias towards LT solutions. The substitution rates estimated from runs with only deep date priors were consistently lower than those estimated when the more recent priors were included (e.g., 0.14 vs 0.71 for the first codon positions) and these differences were statistically significant. Unfortunately, Beast does not yet allow rates to vary along a lineage through time; however, in the present study implausible Oligo-Miocene divergence dates were estimated for the divergence of the higher taxa (e.g., Hydrobiidae and Amnicolidae) if recent priors were included in the calibration (regardless of the priors on clock rates - various subsets, types and ranges of priors were tested). Substitution rates in pomatiopsid lineages appear to have accelerated at some time prior to the Miocene; this unfortunately dictated an arbitrary division of the dating analyses between those having an *a priori* expectation of being pre-Miocene (the “deep divergence” runs) and those expected to be Miocene and post-Miocene (the “recent” runs). The overall rate for the *cox*1 data (all codon positions) is 2.13%; this compares well with published rates for molluscan *cox*1 molecular clocks; for example 1.21% for Arcidae (Bivalvia) [89], and 1.18% for freshwater tropical Ampullariidae [90]. Similarly, the rate of 1.29% for *rrn*L in Table 3, is comparable with those of 1.10% for triculine snails over the early Pleistocene [91], and 0.1%–2.2% for invertebrates in general [92].

**Table 3:** Results of a Bayesian estimation of nucleotide substitution rates

**Parameter**	**Mean ± S.E.**	**ESS**	**95% HPD (lower – upper)**
-ln (likelihood) Deep divergences	−10921.7241 ± 0.201	2463.419	−10921.3095 – –10902.1831
-ln (likelihood) Most recent divergences	−10924.1375 ± 0.1375	5406	−10944.6406 – –10905.5067
Mean substitution rate (*cox1*) First codons	0.70605 ± 0.0031356	4740.8697	0.37368 – 1.1606
*Mean substitution rate (*cox*1) First codons*	*0.14204 ± 0.0014602*	*2164.1316*	*0.057033 – 0.2659*
Mean substitution rate (*cox1*) Second codons	0.087271 ± 0.00046307	4523.76	0.036775 - 0.14957
*Mean substitution rate (*cox*1) Second codons*	*0.017227 ± 0.00016606*	*2703*	*0.0055299 – 0.033703*
Mean substitution rate (*cox1*) Third codons	25.54 ± 0.06864	4361.6091	17.16 – 34.53
*Mean substitution rate (*cox*1) Third codons*	*7.2543 ± 0.079013*	*1418.9629*	*2.7901 – 13.05*
*Mean substitution rate (*cox*1) All Sites*	*2.1253 ± 0.033205*	*818.4771*	*0.8405 – 3.9242*
Mean substitution rate (*rrn*L)	1.2869 ± 0.0038937	3539.2582	0.79066 – 1.854
*Mean substitution rate (*rrn*L)*	*0.27031 ± 0.0021339*	*2703*	*0.12723 – 0.47463*
Mean substitution rate indels	0.73603 ± 0.0033437	3539.2582	0.37765 – 1.094
*Mean substitution rate indels*	0.14923 ± 0.0012716	2703	0.06371 – 0.26997

The mean substitution rate is expressed per 100 bases per Ma. ESS, effective sample size (i.e., size corrected for auto-correlation); HPD, the 95% highest posterior probability density (equivalent to a confidence interval); SE, standard error of the mean. The substitution rates are given for the more recent divergences runs followed by the data for the deep divergences runs in italics. The rate for *cox1* all sites was obtained from the initial test runs.

Beast was used to estimate TMRCAs for clades within the hydrobioid phylogeny, the results of this are given in Table 4. In this table the TMRCAs are compared with their predicted values according to the Tibet and the Hunan hypotheses. If the prediction lies entirely within the HPD of the date estimated after observation of the data the corresponding hypothesis was considered to be fully supported by the results. Table 4 indicates that the results of the molecular dating analysis supported the Hunan hypotheses in 9 out of 13 comparisons, whereas the Tibet hypothesis was only supported in one comparison. Four TMRCAs were inconsistent with both hypotheses. It must be noted that the Hunan hypothesis was more detailed than the Tibet hypothesis and so the former would be likely to be consistent with more TMRCAs; however, overall it is clear that the time scale inferred from the data is in better agreement with the Hunan hypothesis.

**Table 4:** Results of a Bayesian estimation of divergence times

**Parameter [node on tree]**	**Mean ± S.E.**	**ESS**	**95% HPD (lower – upper)**	**Relevant predictions**	**Predictions supported**	**Hypothesis**
**tmrca (ingroup) [0]**	114.533 ± 0.6187	2703	52.5946 – 176.4489	H1, T1	≤ H1, T1	Both
**tmrca (Pomatiopsidae) [**[2]**]**	110.760 ± 0.5864	2703	51.8809 – 170.102	H2, T2	H2	Hunan
**tmrca (Pomatiopsinae) [**[10]**]**	59.0213 ± 0.5583	2195	16.9746 – 111.8963	H3, T4	≤ H3	Hunan
**tmrca ( *Oncomelania *) [**[11]**]**	22.4588 ± 0.2018	2703	15.4905 – 34.7171	H4	≤ H4	Hunan
tmrca (Sunda) [12]	7.925 ± 0.048796	2703	3.9652 – 13.1893	H9, T5	≥ H9	Hunan
tmrca (Thai) [14]	5.963 ± 0.020944	3668.2865	3.621 – 8.469	H11, T7	≥ H11, ≤T7	Both
tmrca (North_Sunda) [18]	5.1588 ± 0.01804	3564.0486	3.1019 – 7.1554	H8, T5, T7	H8, ≤T7	Both
tmrca (Yunnan) [25]	4.1509 ± 0.01424	4270.9393	2.4844 – 5.9712	H14		N
tmrca (Malaya) [26]	3.5555 ± 0.01417	2337.4983	2.3954 – 5.0629	H13, T7	≥ H13, ≤ T7	Both
tmrca (Mekong) [41]	2.3658 ± 0.00790	4150.45	1.4563 – 3.3614	H12, T7	H12	Hunan
tmrca (Khorat) [42]	0.8679 ± 0.00140	5406	0.6755 – 1.0775	H15	H15	Hunan
tmrca (Jullieniini) [43]	0.7847 ± 0.00363	5406	0.3492 – 1.3228	H12, T7		N
tmrca (Lao) [63]	0.3561 ± 0.00154	4787.2537	0.181 – 0.569	H8, T5		N

**Figure 6:**
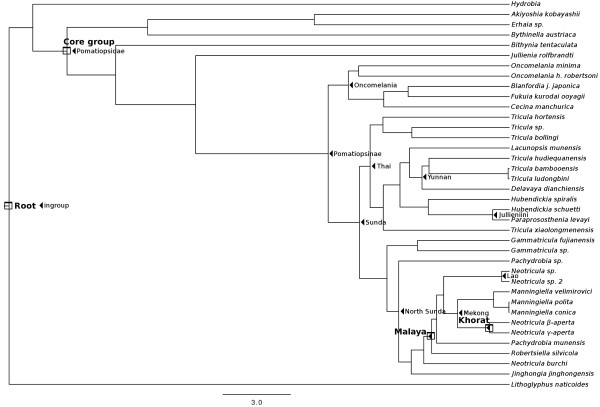
**Phylogenetic tree generated by Beast showing callibration points and priors.** A maximum clade credibility tree estimated by Beast with deep dating priors (see Hypotheses testing in Methods). Callibration priors are indicated by square boxes and estimated TMRCAs by black triangles. The names applied to the priors refer to Tables 2 and 4.

Time estimates are given in millions of years (Ma) for nodes representing the most recent common ancestor of relevant clades. ESS, effective sample size (i.e., size corrected for auto-correlation); HPD, the 95% highest posterior probability density (equivalent to a confidence interval); SE, standard error of the mean; TMRCA, time to most recent common ancestor of the taxa in the clade. Relevant predictions refers to the numbered predictions in Table 1 for each hypothesis (prefix H refers to the Hunan hypothesis and T refers to the Tibet hypothesis). Entries in bold were estimated by runs for deep divergences and other entries were estimated by runs for more recent divergences. A prediction is supported if it is within the HPD of the date estimated after observation of the data. The final column lists which of the two hypotheses is best supported by the data and analysis (or N in the case that neither hypothesis is consistent with, or relevant to, the parameter estimate, or “Both” where both are consistent). Inequalities symbols indicate where the estimated tmrca is slightly too low (<) or too high (>) but borderline with one or both hypotheses. The tree nodes refer to the phylogeny in Figure 6 and are counted from the root (node 0).

## Discussion

### Phylogeography

#### Origin of the pomatiopsidae

The results of the hypothesis tests indicated that neither the Tibet nor the Hunan hypothesis was consistent with the estimated phylogeny; however, the Hunan hypothesis was mostly consistent with the estimated times of divergence within the pomatiopsid radiation. Past work, based on fossil record, morphology of extant taxa, and phylogeography (Tibet origin) suggested that the major burst of radiation in the Triculinae occurred late Miocene (12–5 Ma) [15]. In contrast, the present analyses indicate that rapid cladogensis among the Triculinae occurred between TMRCAs Thai and Mekong (Figure 6, Table 4), i.e., 6–2.4 Ma.

Table 4 indicates that the Pomatiopsidae diverged from the Amnicolidae around 111 Ma; this is consistent with the break up of the minor Gondwanan continent (which included Australasia) around 132 Ma and rafting of pomatiopsine-like proto-Pomatiopsidae on these Gondwanan fragments, with subsequent island hopping northwards (and some (tectonic) rafting) and radiation of marine pomatiopsines along a Borneo-Philippines tract and also to the southern continents (South Africa and South America). The estimated date is less consistent with a divergence prior to the break up of Pangaea and rafting of proto-pomatiopsids (resembling Triculinae) and Asian amnicolids on the Indian craton (around 180 Ma); however, the upper bound of the HPD for this estimate was 170.102 Ma, which is close to 180 Ma. Consequently, this date alone is not sufficient to rule out any hypothesis.

The common ancestor of the Triculinae and Pomatiopsinae was estimated to have occurred around 59 Ma (Table 4). This taxon may have inhabited Eastern Gondwana (Australia) after the loss of India/Madagascar, with later island hopping from Borneo and Sulawesi along the extensive island complex existing in the region during the Eocene-Oligocene. These snails could have then given rise to the antecedent Triculinae when they reached China, and Pomatiopsinae on the Japanese islands. The Tibet hypothesis would require the Pomatiopsinae to be derived from the Triculinae or the former to have crossed the uplifting Himalaya (something which for the extant Pomatiopsinae show little adaptation); the southern margin of the Qinghai-Tibet Plateau achieved present day levels 22–15 Ma. In addition, extensive and semi-continuous volcanism, 45–26 Ma and around 20 Ma [93], in the Himalayas makes trans-Himalayan dispersal tracts even more unlikely. Volcanism along the Central Volcanic Arc also would have restricted migration across Myanmar even until the Pleistocene [94].

#### Radiation of the pomatiopsinae

The TMRCA estimates next suggested very little divergence among Pomatiopsidae (at least among lineages with extant representatives) until around 22 Ma, with high rates of cladogenesis 8 – 2 Ma (Table 4). During the early Miocene the Japan Sea opened and the Japanese islands separated from China. Many of the Japanese islands underwent a cycle of emergence, submergence and re-emergence (18–16 Ma) [95]. The regional climate at that time also became warm and humid [52,96]. These events could have driven the divergence of amphibious and terrestrial Pomatiopsinae in Japan. Figure 6 and Table 4 suggest that this occurred around 22 Ma, with *Oncomelania* shown to have appeared prior to the other Japanese Pomatiopsinae; this would have allowed ancestors of Chinese *Oncomelania* (*O. hupensis*) to have colonized mainland China before land connections were broken (around 15 Ma [54]). *O. minima* and the terrestrial pomatiopsines are not found in China. The Pomatiopsinae are also absent from Korea (which is unexpected if they colonized Japan from China), possibly because deep marine basins have isolated the Korean peninsula since the Palaeogene [97].

#### Divergence of the triculinae

The divergence of the Pachydrobiini from a clade comprising the Triculini and Jullieniini is estimated to have occurred around 8 Ma (Table 4, Sunda). This date coincides with the attainment of present day elevations across the whole Qinghai-Tibet Plateau [98] and global changes in climate, with the Himalaya obstructing global air circulation and the predominance of a monsoon climate in the region [99]. Most Triculini are restricted to primary streams (usually from springs) in highland areas, whereas almost all Pachydrobiini live in larger streams and rivers. The isolation of spring-dwelling highland taxa in uplifting regions of Southwest China and the more arid climate could have triggered the divergence of the proto-Triculinae, with the invasion of larger rivers and streams. From this point onwards a rapid acceleration in triculine radiation and cladogenesis is observed (Figure 6); the acceleration among the Triculini could have been driven by deepening of the valleys in which they live (i.e., increased isolation) and that among the Pachydrobiini by the greater variety of new habitats and area covered following the invasion of rivers. The next divergence event is that of the *Tricula* of northern Thailand (including *T. hortensis* of Sichuan) from a clade comprised of Jullieniini and the Yunnan Triculini; this event is dated at around 6 Ma (Thai, Table 4). Table 1 links this event (under the Hunan hypothesis) to Late-Pliocene (3.4 Ma) block faulting which may have induced several course changes along the proto-Mekong river [2] and the break up of connexions between Yunnan/Guangxi China and northern Thailand via the extended Mekong-Ping river, and flow reversals [20] in Northwest Sundaland. The estimated date of 6 Ma, however, is more closely aligned with the second major uplift of the Himalaya (7.5-6.5 Ma, [49]), which would have also led to uplift along the Shan formation and other ranges acting as barriers between China and Sundaland. Uplift of northern Thailand would also have accelerated and would have isolated taxa there from those radiating in the lower Mekong river. The next divergence, dated at around 5 Ma, concerns the Pachydrobiini, with all other Pachydrobiini (mostly Sundaland and Mekong river taxa) arising from a lineage now represented by *Gammatricula* (of Guangxi/Fujian China) (North Sunda, Table 4). Table 1 (Hunan hypothesis) associates this event with the loss of the Red river corridor between southern China (Hunan/Guangxi) and Northeast Sundaland [20] at around 4.4 Ma [55].

#### The sundaland radiation

**Figure 7:**
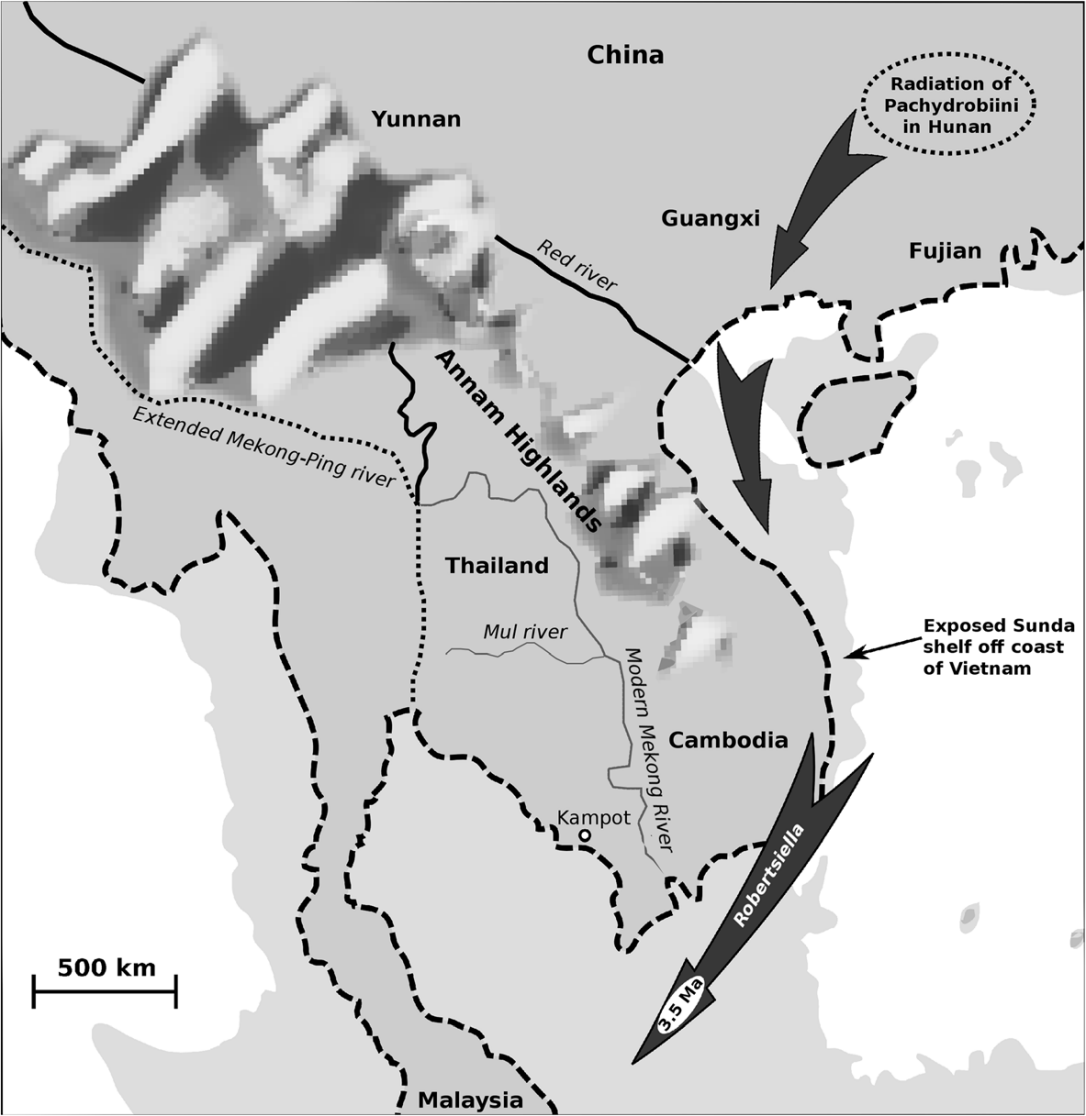
**The radiation of Pachydrobiini into Sundaland.** Semi-schematic depicting a possible late Miocene dispersal route for *Robertsiella-Guoia* clade Pachydrobiini diverging in Hunan to enter Sundaland via now extinct river systems draining the, now submarine, Sunda shelf. This model allows for the observed independent and heterochronous colonization of Sundaland by *Neotricula* and *Robertsiella*. Present day coastlines are shown by thick broken lines, the modern Mul and Mekong rivers are indicated as thin grey lines and the Miocene extended Mekong-Ping river is shown by a dotted black line. The extent of the Miocene coastline beyond that of the present is indicated in light grey. Geological features and coastlines approximate.

The phylogeny in Figure 6 then shows a series of Plio-Pleistocene divergences among minor taxonomic groups. The divergence of *Delavaya* of Dianchi lake (Yunnan) from the spring-dwelling *Tricula* of Yunnan is estimated to have occurred around 4 Ma; this appears to correspond with the creation of Dianchi lake in the Pliocene, some time prior to 3.4 Ma [100]. Table 4 (Malaya) shows that *Robertisiella* diverged from *Neotricula aperta* and other Mekong river Pachydrobiini around an estimated 3.5 Ma. Table 1 links this divergence to events isolating the Pahang river system of West Malaysia from the extended Mekong river due to river course changes and rising sea levels which flooded the Sunda shelf (2.6-0.8 Ma), the Pliocene Mekong river flowed directly from southern Laos, across Cambodia (via the Tonle Sap of today) to Kampot and then into Pahang drainage of Malaysia (Figure 7) [59,60]. More recent evidence suggests a particularly warm and generally humid global climate between 3.56 and 3.4 Ma [101]; this would certainly have flooded the Sunda shelf and isolated Malaysian *Robertsiella* from the Mekong river pachydrobiinine radiation. The divergence of *Robertsiella* from *Neotricula* appears to predate that of *Schistosoma mekongi* from *S. malayensis*, which was dated at around 1.3 Ma [60], although the polarity of *Schistosoma* does appear to match that of their intermediate hosts (i.e., *Robertsiella* appears to have arisen first – Davis proposed that this genus arose as part of the Hunan pachydrobiinine radiation [8]). The divergence of *Robertsiella* could also be associated with the accelerated uplift of the eastern edge of the Qinghai-Tibet Plateau at 3.4 Ma [56], affecting Sichuan, Yunnan and Sundaland along the eastern margin of the Sibamasu block. The greater affinity of *Robertsiella* with taxa of North Sundaland and Yunnan (*N. burchi* and *Jinghongia*) supports earlier studies that suggested a direct tract of dispersal of *Robertsiella* from Hunan to the proto-Mekong river of Cambodia, across the exposed Sunda shelf off the modern coast of Vietnam [53] (Figure 7); thus explaining why *Robertsiella* is not derived from the lower Mekong Pachydrobiini clade and suggesting that *Neotricula aperta* arrived in Cambodia via a slightly different drainage system.

The next major event dated was the divergence of *Manningiella* and *Neotricula* in the lower Mekong Basin, which was estimated at around 2.4 Ma (Table 4, Mekong). This event coincides with the end of a global cooling period (2.7-2.5 Ma) [32], and a significant Asian monsoon intensification occurred prior to the major glaciation at around 2.7 Ma, which triggered an extremely arid 1.4 Ma interval [102]. Such climatic events could have driven the divergence of proto-Mekong river Triculinae as they re-encountered more shallow water, stream-like habitats. Finally, the divergence of the beta and gamma strains of *Neotricula aperta* is dated at around 0.87 Ma (Khorat, Table 4); this date agrees well with the predictions of earlier studies (Table 1, H15). The divergence of *N. aperta* was considered to be driven by events linked to the final surge of the Himalayan uplift [49], such as the tilting of the Khorat Basin, reversal of the flow of the Mul-river, and volcanism in southern Laos which temporarily separated the lower Mul river from the Mekong [19].

### Taxonomic considerations

#### Erhaiini and jullieniini

The phylogenies in Figures 4 and 5 both indicate similar phylogenetic affinities, which help to clarify the taxonomy of the Asian “hydrobioids”. The taxon resembling *Erhaia*, which was collected in Guangxi Province, China, is seen to form an amnicolid clade together with *Akiyoshia* of Japan and the European *Bythinella austriaca*; this further supports the decision of other authors [17,18] to transfer all Pseudobythinellini/Erhaiini to the Amnicolidae. The position of *Erhaia* was uncertain because although this taxon possesses a *Hydrobia-*like central tooth (as in Amnicolidae) it shows closure of the ventral channel to form a spermathecal-duct like structure (as in Pomatiopsidae) and the penial hold-fast is absent [15]. *Erhaia* is known from Fujian, Hubei, Hunan, Sichuan and to a lesser extent, Yunnan, China; the finding of *Erhaia* in Guangxi, though expected (Guangxi lies between Fujian and Yunnan), extends the known range of this genus.

The position of *Jullienia rolfbrandti* at the base of the pomatiopsid clade (Figures 4, 5 and 6) is unexpected. In the tree inferred by Beast (Figures 5 and 6) all other Jullieniini form a distinct clade within the Triculinae and arise from the base of the West China Triculini lineage. In the phylogeny estimated by RAxML, the Jullieniini do occur on (monophyletic) adjacent branches with *Jullienia* the most basal, and all Jullieniini being clustered at the base of the pomatiopsid phylogeny. Consequently, the unexpected position of *Jullienia* on the Beast tree may be a failure of the analysis to land upon a correctly resolved tree; however, it is equally likely that the Jullieniini is an artificial group. The Jullieniini were envisaged as having diverged from the lineage leading to the Pachydrobiini, Triculini and *Lacunopsis* during the Miocene; they were considered to be united by a modification of the central tooth and adaptations of the female reproductive system (e.g., the oviduct circle complex), and another derived character state of the Triculinae, that of large shell size [10]. The misidentification of specimens or mis-labelling of DNA extracts in the laboratory is unlikely because there were only samples of Triculinae in the laboratory at that time and no other genus from the collecting site has a shell, radula and gonad resembling a *Jullienia* sp. that is not Triculinae.

#### The Triculini and their relationships with other triculine taxa

Turning next to the Triculini, it can be seen that *Tricula* is monophyletic in neither the Bayesian (Beast) nor ML (RAxML) phylogenies (Figures 4 and 6). The northern Thai *Tricula* and *Tricula hortensis* are found at the root of the Triculini. This Thai clade forms a bifurcation with a clade comprising the Jullieniini (at the root) and the West China (Yunnan) Triculini, which include *Tricula bambooensis, T. hudiequanensis, T. ludongbini* and *Delavaya*, with *Lacunopsis* at the base. The remaining *Tricula* species, *T. xiaolongmenensis*, is found at the root of the Triculini + Jullieniini clade. Even allowing the possibility of a small degree of systematic error affecting the exact distribution among clades, the genus *Tricula* is clearly not monophyletic (the credibility of the aforementioned clade is 0.87, which is greater than the 0.81 for the monophyly of the Triculinae, Figure 5). Earlier morphological studies placed the Chinese *Tricula* in a clade closely associated with *Delavaya* and *Lacunopsis*, with the remaining *Tricula* (including the type species *T. montana*) in a clade at the base of the Triculini and closest (among Triculini) to *Hubendickia*. The present results appear to support this division which was recognized in a series of remarkably detailed earlier morphological studies [8]. Although it is not possible to obtain a sample of *Tricula montana* (due to India’s bio-resources protection policy), the basal position of *T. montana* in the cladogram estimated by Davis et al., [1], suggests that the northern Thai species (including *T. bollingi*), and possibly also *T. hortensis,* and *T. xiaolongmenensis* are *Tricula sensu stricto*, whereas, the Yunnan species should not be regarded as *Tricula.* The Yunnan “*Tricula*” are congeners of *Delavaya*, whether or not they can all be classed as *Delavaya spp.* would depend on a detailed re-examination of the anatomy of *T. hudiequanensis*. In Figure 4, the ML phylogeny, *T. hortensis* is found at the base of a poorly supported (8%) clade including the Pachydrobiini and Pomatiopsinae! This is most likely a failure of phylogenetic estimation by direct ML; however, it is worth considering that previous authors noted the difficulties of determining the affinities of this taxon – which was described as difficult to place within the Pomatiopsidae [23], or as showing character states common to both Triculinae and Pomatiopsinae. The inclusion of *Lacunopsis* at the root of the Yunnan Triculini clade agrees with the conclusions of morphological studies that *Lacunopsis* arose from the Triculini and is close to *Delavaya*[8]. Finally, the relationship needs to be considered between *T. bollingi* and a species of *Tricula* found in a spring only 11.2 km distant. The new taxon is most likely a species distinct from *T. bollingi* because, when compared with other sister species of *Tricula* (e.g., *T. bambooensis* and *T. ludongbini*) the divergence between this new taxon and *T. bollingi* is much greater. The two taxa also appear to have diverged around 5 Ma; this suggests that the two have very different histories, for example, the new taxon may have originated locally, whereas *T. bollingi* could have been introduced from China or Myanmar (for example on religious artifacts transported by monks – the collecting site for *T. bollingi* was downstream of a large temple). *Tricula bollingi* is also known from Yunnan, China [1]. Additional file 4: Table S1 lists taxa highlighted for revision and suggested changes.

Considering next the relationships of the remaining Jullieniini, the direct ML trees (PAUP* Additional file 1: Figure S1 and RaXML Figure 4) and the Bayesian estimated phylogenies all show these taxa as arising at the root of the clade leading to the Triculini; however, in all these trees these jullieniinines do not occur in a cohesive monophyletic clade and the Triculini are at the base of the Pomatiopsidae, whereas in the Beast estimated tree, the Triculini form a bifurcation with the Pachydrobiini in a monophyletic Triculinae clade, and these Jullieniini form a monophyletic clade at the base of the West China Triculini (Figure 5). *Hubendickia spiralis* lies at the root of this clade, with *Hubendickia schuetti* and *Paraprososthenia levayi* as sister taxa; this is well supported (credibilities 0.99 and 1.0, respectively). The implication is that *Hubendickia schuetti* is *Paraprososthenia*. Indeed, this taxon was first described as *Paraprososthenia*[12] and was later transferred to *Hubendickia* because of its ovate-pyriform aperture. The tribe Jullieniini is characterized by a specialized central tooth (excluding some *Hubendickia*) and elongation (*Hubendickia*) to extreme elongation (*Paraprososthenia, Jullienia, Hydrorissoia,* and *Pachydrobiella*) of the seminal receptacle. Increasing degrees of elongation, reflexion or coiling of different parts of the vas deferens are also seen as a characteristic of increasingly derived jullieniinine genera, the extreme being seen in *Jullienia and Hydrorissoia*[43]. These adaptations of the reproductive system have been considered as responses to mating in the early flood period of the lower Mekong river, which is the time when these taxa complete their life-cycles [10,43,103]. In view of this it is possible that the character states serving as synapomorphies uniting the Jullieniini are simply due to convergence; thus, the apparent affinity of *schuetti* with species of *Hubendickia* may simply reflect a similar microhabitat and rapid radiation in the evolving Pleistocene lower Mekong river (this divergence was dated to around 0.8 Ma, Jullieniini, Table 4). Convergence may also explain the failure of *Jullienia rolfbrandti* to cluster with the other Jullieniini in this study.

#### Taxonomy of the pachydrobiini

The Pachydrobiini show a near isochronous burst of divergence events with that seen among the Triculini, suggesting that both lineages responded to the same event (probably the second major uplift of the Himalaya and Sibamasu orogeny, see above). In the phylogenies estimated by ML and Bayesian approaches *Gammatricula* is seen to occur at the root of the Pachydrobiini. In the Beast (and PAUP*) phylogeny the two *Gammatricula* spp. form a monophyletic clade, in the RAxML tree they co-occur with *Jinghongia* and the Vietnamese taxon resembling *Pachydrobia*. The observation is interesting because *Gammatricula* was described as a genus showing character states that were overall derived among Pachydrobiini [8]. The Beast phylogeny shows that the taxon from northern Vietnam (*Pachydrobia* sp.) lies at the root of the Pachydrobiini, just above the *Gammatricula* clade (or inside it according to ML), and therefore is unlikely to be *Pachydrobia*. Snails described as *Vietricula* species have been described from Cao Bang in northern Vietnam [30], from a site approximately 200 km East of the collection locality for the taxon collected in the present study. The descriptions in the 2012 study appear to be based on shell character states and so the possibility that these snails were derived from early Pliocene Chinese *Gammatricula* in the nearby provinces of Yunnan and Guangxi cannot be excluded.

*Jinhongia,* which is found in the middle Mekong river in Yunnan, China, is found at the base of the Sundaland Pachydrobiini clade; this sugests that around 4 Ma taxa from Guangxi/Yunnan entered Sundaland from the North and began a rapid radiation southwards in the lower Mekong river. The southwards radiation is in accordance with the Tibet hypothesis as originally proposed [10], although the time scale is different. The next extant taxon in this southwards radiation is *Neotricula burchi.* Regardless of the phylogenetic method, the present data indicated that *Neotricula* was not monophyletic because species of *Manningiella, Pachydrobia* and *Robertsiella* are found with the *Neotricula* clade. The type for the genus *Neotricula* is *N. aperta* (assignation [104]), so that *Neotricula burchi* should be placed in a new genus (or in *Jinhongia –* additional samples of *Robertsiella* are needed to decide this). The three species of *Manningiella* sampled form a well supported (Figure 5, 0.98) clade with *N. aperta*; therefore it is possible that these are in fact species of *Neotricula*. Additional, sampling (of loci and species) and morphological work is required on *Manningiella* and the two taxa from northern Thailand and central Laos (here denoted as *Neotricula* sp. and *Neotricula* sp. 2) to determine if these taxa are indeed all *Neotricula* spp. The question is one of particular importance because *Neotricula aperta* acts as intermediate host for *Schistosoma mekongi* and it is clearly very closely related to taxa described as *Manningiella.* It should be mentioned that most, perhaps all, species originally assigned to *Manningiella* by [105] have been transferred to other genera (even including non-pachydrobiinine genera, such as *Hubendickia*), e.g., *M. conica* and *M. expansa* were assigned to a new genus, *Halewisia,* on the observation that the spermathecal duct runs directly into the duct of the bursa copulatrix in an anterior position (in other Triculini it enters in a posterior position), the sperm duct does not form a common sperm duct with the duct of the seminal receptacle (so resembling *Pachydrobia* and *Tricula*), and shells are smooth, ovate-conic, with expanded inner lip [10]. In the present study we used the name *Manningiella* because the detailed descriptions of taxa such as *polita* and *velimirovici* refer to *Manningiella,* because *Halewisia* is not in common usage, and because there are no detailed accounts of the anatomies of several species assigned to the nominal taxon *Halewisia*. Nevertheless, it should be noted that the species *polita* and *conica* listed here as *Manningiella* have been referred to *Halewisia* in some works [10].

## Conclusions

The present study has demonstrated that the idea of an Australasian origin for the Pomatiopsidae, followed by an East to West radiation is a plausible alternative to a hypothesis of rafting of Pomatiopsidae on the Indian craton, with divergence in the Himalaya and a westwards colonization of Sundaland and China in the early Miocence. The findings provide support for a Hunan origin for the Triculinae, no later than around 8 Ma. The radiation the Pomatiopsinae is seen to begin some 14.5 Ma prior to that of the Triculinae; this suggests that the former diverged first (possibly in Japan) and the Triculinae diverged in China much later and probably from pomatiopsine stock. The scenario that both sub-families diverged in Tibet as they colonized the upper Yangtze and Mekong rivers, respectively, is less probable in the light of the current data. The results confirm the rapid radiation and burst of cladogenesis in the Triculinae, as postulated by previous authors [15,21,64], and suggest that the acceleration began around 6 Ma. The molecular dating indicates that the radiation of these snails was driven first by the uplift of the Himalaya and onset of a monsoon system, and subsequently by late-pliocene global warming.

The analyses have helped confirm the status of *Erhaia* (formerly tribe Erhaiini) as Amnicolidae rather than Pomatiopsinae: Pseudobythinellini as previously thought and have extended the known range of *Erhaia* in China. The phylogenetic estimations demonstrated that the genera *Tricula* and *Neotricula* are not monophyletic and that the Jullieniini maybe an artificial taxon based on convergent responses of the hydrobioid female reproductive anatomy to life in the faster and more extreme flow regime of the lower Mekong river. The study showed that several species assigned to *Halewisia/Manningiella* are very closely related to *Neotricula aperta* and this is important for the transmission of Mekong schistosomiasis because *N. aperta* is the only known intermediate host for *Schistosoma mekongi*. The intermediate host of *Schistosoma malayensis*, *Robertsiella,* was also shown to have a possibly independent evolutionary history from *Neotricula aperta,* with a more direct entry into Cambodia via now extinct and submarine river systems linking Hunan-Vietnam and Cambodia; this also has implications for the radiation of *Schistosoma*. The work also supported the conclusions of George Davis, based on intricate and remarkably detailed anatomical studies of the Triculinae, that there were divisions within the genus *Tricula* and of the origin of the Lacunopsini from the stem of the Triculini clade.

The present study has also raised several new questions which require further study. These include additional work on *Pachydrobia, Robertsiella* and *Jinghongia* with the inclusion of more species (except *Jinghongia*) and more loci, so that the generic status of *Neotricula burchi* can be elucidated. Surveys in Fujian and Guangxi, China, are required to detect further species of *Gammatricula* or *Vietricula,* to determine the genera of these taxa and their phylogeographical history. Much more anatomical work is required for *Halewisia*, to determine which species should be assigned to this genus and the extent of affinities with *Neotricula aperta*. Renewed parasitological investigations are also required in this context, past investigations suggested that miracidia of *S. mekongi* can locate and penetrate *Manningiella expansa* but that the snails died before the sporocysts would have become patent [106]. In later experiments 12% of *Manningiella conica* exposed to *S. mekongi* were observed to shed cercariae, which is a rate very close to that of similarly exposed *N. aperta*[107]. Aside from this, the transmission of *Schistosoma* appears limited to 3 clades, scattered across the phylogeny, with a most recent common ancestor around the Paleocene; this suggests that the evolutionary relationship between these snails and *Schistosoma* is more like loose phylogenetic tracking, rather than co-evolutionary. The finding has implications for future studies of these host-parasite relationships and prediction of the long-term impacts of snail control in regional schistosomiasis. The present study included 16 genera of Pomatiopsidae but this represents only 4 of 9 Pomatiopsinae, 3 of 5 Triculini, 3 of 9 Jullieniini, and 6 of 9 Pachydrobiini genera (this excludes those genera of uncertain affinity or data deficient, e.g. *Saduniella, Vietricula*). Clearly there is much scope for future studies with more extensive taxon sampling and, if taxa can be re-discovered (where necessary) and re-sampled, the inclusion of additional loci in the phylogenetic analyses. It is hoped that the present study has provided the first step in solving the taxonomic confusion and lack of data surrounding this at risk, highly diverse and medically important group of freshwater snails.

## Methods

### Sampling

**Figure 8:**
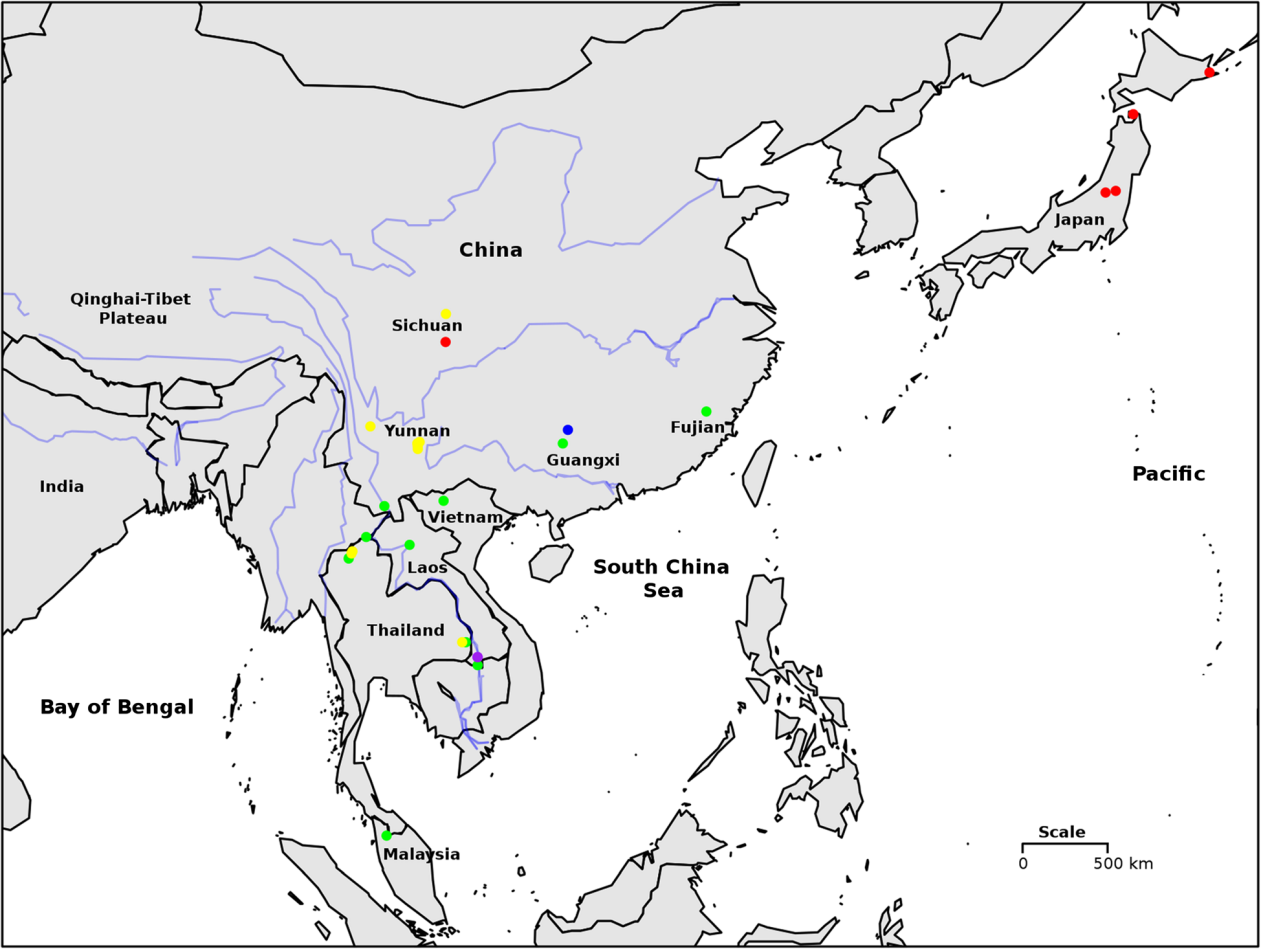
**Sampling localities for the present study.** Map to show the collecting localities or origin of taxa (in the case of data from the GenBank) used in the present study. Coloured spots indicate the samples for each main taxonomic group: Erhaiini, blue; Jullieniini, purple; Pachydrobiini, green; Pomatiopsinae, red; Triculini, yellow. Map generated using the R packages maps and mapdata [6,7] and the data in Table 5.

The field sampling covered 4 countries, 3 provinces of China, 9 river/drainage systems and 11 genera, with DNA for 6 genera (16 species) sequenced for the first time, and 21 species sequenced for the first time at the loci sampled (Figure 8). Additional data sourced from the GenBank extended the study to include 38 taxa, encompassing 17 genera from 9 countries and 19 drainage systems. Table 5 gives details of taxa sampled, Genbank accession numbers and collection localities for all sources of sequence data. All field collected specimens were identified by general shell form, radular characters, ecological habit, examination of head-foot and operculum, and by gross dissection of pallial and reproductive structures, with reference to detailed published descriptions [8,10,13-15,21,59,104-106,110-115]. Samples for DNA extraction were fixed in the field directly in 100% ethanol, with a sub sample of each taxon fixed in 10% neutral formalin for morphological study.

**Table 5:** Taxonomic and collection details of the taxa included in the phylogenetic reconstructions

**Taxon**	**Tribe**	**Region**	**Water system**	**Locality**	***GPS***	**Accession no.**
*Akiyoshia kobayashii*	Amnicolinae	Japan: Hokkaido	Serikawa River	Taga, Shiga	Not given	*GenBank: AB611823, GenBank: AB611822*[28]
*Bythinella austriaca*	Amnicolinae	Poland: Krakow	Vistula	Dolina Eliaszòwki	Not given	*GenBank: AF445333, GenBank: AF445342*[44]
*Erhaia sp.*	Amnicolinae	China: Guangxi	Xiangjiang River	Quanzhou	25.73790, 110.71881	***GenBank: KC832701, GenBank: KC832722***
*Bithynia tentaculata*	Bithyniidae	Germany	Spree	Müggelsee (near Berlin)	Not given	*GenBank: AF445334, GenBank: AF445344*[44]
*Hydrobia acuta/Hydrobia sp*	Hydrobiidae	Italy/?	Tyrrhenian Sea	Toscana/Not given	Not given	*GenBank:* AY010324 [108]; *GenBank: AF212898*[109]
*Hubendickia schuetti*	Jullieniini	Lao PDR	Mekong River	Ban Xieng-Wang Village	14.11764, 105.85620	***GenBank: KC832688, GenBank: KC832709***
*Hubendickia spiralis*	Jullieniini	Lao PDR	Mekong River	Ban Xieng-Wang Village	14.11764, 105.85620	***GenBank: KC832689, GenBank: KC832710***
*Jullienia rolfbrandti*	Jullieniini	Lao PDR	Mekong River	Ban Xieng-Wang Village	14.11764, 105.85620	***GenBank: KC832697, GenBank: KC832718***
*Manningiella conica*	Pachydrobiini	Lao PDR	Mekong River	Ban Hat-Xai-Kuhn	14.12056, 105.86586	***GenBank: KC832698, GenBank: KC832719***
*Manningiella polita*	Pachydrobiini	Thailand	Mul River	Phibun Mangsahan	15.25554, 105.23343	***GenBank: KC832694, GenBank: KC832715***
*Manningiella velimirovici*	Pachydrobiini	Lao PDR	Mekong River	Ban Hat-Xai-Kuhn	14.12056, 105.86586	***GenBank: KC832695, GenBank: KC832716***
*Paraprososthenia levayi*	Jullieniini	Lao PDR	Mekong River	Ban Hat-Xai-Kuhn	14.12056, 105.86586	***GenBank: KC832687, GenBank: KC832708***
*Lithoglyphus naticoides*	Lithoglyphinae	Germany	Spree	Spree (near Berlin)	Not given	*GenBank: AF445332, GenBank: AF445341*[44]
*Gammatricula fujianensis*	Pachydrobiini	China: Fujian	Jianxi River	Nanping, Lichangken	Not given	*GenBank: AF213342, GenBank: AF212896*[109]
*Gammatricula sp.*	Pachydrobiini	China: Guangxi	Lijiang River	Guanyan	25.06578, 110.44598	***GenBank: KC832703, GenBank: KC832724***
*Jinghongia jinghongensis*	Pachydrobiini	China: Yunnan	Mekong River	Xishuangbanna	21.96796, 100.85190	***GenBank: KC832707, GenBank: KC832728***
*Neotricula burchi*	Pachydrobiini	Thailand	Ping River	Chiang-Dao Cave	19.39167, 98.93333	*GenBank: AF531542, GenBank: AF531544*[20]
*Neotricula sp.*	Pachydrobiini	Lao PDR	Mekong River	Pak Ou	20.05234,102.20804	***GenBank: KC832704, GenBank: KC832725***
*Neotricula sp.2*	Pachydrobiini	Thailand	Mekong River	Tachileik (Myanmar)	20.43988, 99.87514	***GenBank: KC832706, GenBank: KC832727***
Neotricula β-aperta*	Pachydrobiini	Thailand	Mul River	Phibun Mangsahan	15.25554, 105.23343	*GenBank:AF188213-6, GenBank:EU306205-8*[19,60]
Neotricula γ-aperta*	Pachydrobiini	Lao PDR	Mekong River	Ban Hat-Xai-Kuhn	14.12056, 105.86586	*GenBank: AF531541, GenBank: AF531556*[20]
*Pachydrobia munensis*	Pachydrobiini	Thailand	Mul River	Phibun Mangsahan	15.25554, 105.23343	***GenBank: KC832700, GenBank: KC832721***
*Pachydrobia sp.*	Pachydrobiini	Vietnam	Nam Sai River	Van Ban District	22.22940, 104.03348	***GenBank: KC832690, GenBank: KC832711***
***Robertsiella silvicola***	Pachydrobiini	W. Malaysia	Muda River	Kedah, Baling	5.70833, 100.97083	*GenBank: AF531550, GenBank: AF531548*[20]
*Blanfordia j. japonica*	Pomatiopsini	Japan: Sado	Not applicable	Niigata	Not given	*GenBank: AB611727, GenBank: AB611726*[44]
*Cecina manchurica*	Pomatiopsini	Japan: Hokkaido	Lake Furen	Betsukai	Not given	*GenBank: AB611747, GenBank: AB611746*[28]
*Fukuia kurodai ooyagii*	Pomatiopsini	Japan: Hokkaido	Mabechi River	Mutsu, Aomori	Not given	*GenBank: AB611783, GenBank: AB611782*[28]
Oncomelania h. robertsoni*	Pomatiopsini	China: Sichuan	Nongshui River	Renshou	30.06667, 104.14167	*GenBank: AB531547, GenBank: AB531545*[20]
*Oncomelania minima*	Pomatiopsini	Japan: Sado	Not applicable	Niigata	Not given	*GenBank: AB611795, GenBank: AB611794*[28]
*Delavaya dianchiensis*	Triculini	China: Yunnan	Dianchi Lake	Maoqing Village	24.92652, 102.64193	***GenBank: KC832692, GenBank: KC832713***
*Lacunopsis munensis*	Triculini	Thailand	Mul River	Phibun Mangsahan	15.25554, 105.23343	***GenBank: KC832705, GenBank: KC832726***
*Tricula bambooensis*	Triculini	China: Yunnan	Dianchi Lake	Hei Qiong Duan	25.06881, 102.62275	***GenBank: KC832699, GenBank: KC832720***
***Tricula bollingi***	Triculini	Thailand	Fang River	Fang	19.64167, 99.08889	*GenBank: AF531553, GenBank: AF531551*[20]
***Tricula hortensis***	Triculini	China: Sichuan	Mianyuan River	Han Wang Zhen	31.45133, 104.16184	*GenBank: AB531539, GenBank: AB531554*[20]
*Tricula hudiequanensis*	Triculini	China: Yunnan	Erhai Basin	Taoyuan Village	25.90975, 100.09882	***GenBank: KC832691, GenBank: KC832712***
*Tricula ludongbini*	Triculini	China: Yunnan	Panlong River	Hei Long Tan	25.14001, 102.74516	***GenBank: KC832696, GenBank: KC832717***
*Tricula sp.*	Triculini	Thailand	Mae Fang River	Chai Prakan	19.73229, 99.13568	***GenBank: KC832702, GenBank: KC832723***
*Tricula xiaolongmenensis*	Triculini	China: Yunnan	Dianchi Lake	Haikou Township	24.79423, 102.64819	***GenBank: KC832693, GenBank: KC832714***

Taxon names are those assigned according to morphological character states, prior to any molecular analysis. Taxa in bold indicate known intermediate hosts of *Schistosoma*, a *denotes a snail compatible with a species of *Schistosoma* (all species involved here infect humans, except for *S. sinensium* transmitted by *Tricula bollingi* and *T.* hortensis). GenBank accession numbers (*cox1, rrnL*) in bold indicate new sequence data collected as part of this study. References are given for *rrnL* sequences, and for *cox1* if different. GPS coordinates are latitude, longitude.

### DNA amplification and sequencing

The shells were gently cracked and the bodies removed. The gut and digestive gland were discarded and DNA extracted from the remainder by standard methods [116]. A section of the coding region of the mitochondrial (mt) cytochrome-*c* oxidase subunit I (CO1) gene (*cox*1) was amplified by PCR with the HCO-2198 and LCO-1490 primer pair developed by Folmer et al. (1994) [117], and part of the gene coding for the mt 16S rRNA (*rrn*L) using the primers of [118]. The *cox*1 gene was chosen because it is a popular locus in barcoding projects [119] and showed adequate levels of interspecific variation in past studies of triculine phylogenetics (33% of sites polymorphic across Pomatiopsidae [20]). The mt 16S locus had previously been used successfully to investigate within family relationships among lymnaeids [120] and Pomatiopsidae [3,18,20]. The *rrn*L data were checked for artefacts such as chimeras using Pintail 1.1 [121], with the sequences for *Tricula bollingi* and *Neotricula aperta* used as subjects and *T. hortensis* and *N. burchi* used to verify integrity of the subjects, respectively (these taxa were chosen because they had been sequenced using several pairs of PCR and sequencing primers and had been used in phylogenetic studies of these taxa for many years without any anomalous results). The two loci were also chosen for compatibility with sequence data available for taxa which are no longer known to science as extant (i.e., all known populations are extinct, as in the case of many snail populations around Dianchi Lake) but for which our laboratory, or the GenBank, had *cox*1 and *rrn*L sequences on record.

Total genomic DNA was used as a template for PCR amplification on a Progene thermal cycler (MWG) employing standard PCR conditions [122]. Unincorporated primers and nucleotides were removed from PCR products using the QIAQuick PCR purification kit (QIAGEN). Sequences were determined bidirectionally, directly from the products by thermal-cycle-sequencing using Big Dye fluorescent dye terminators and an ABI 377 automated sequencer (Perkin-Elmer), following procedures recommended by the manufacturers. Sequences were assembled and aligned using Sequencher (version 3.1 Gene Codes Corp. Ann Arbor, Michigan). DNA sequences for both strands were aligned and compared to verify accuracy. Controls without DNA template were included in all PCR runs to exclude any cross-over contamination. A multiple sequence alignment was generated for all taxa for each locus and the *cox*1 and *rrn*L sequences were then concatenated to form a combined data set. Potential outgroup sequences were obtained from the GenBank; these included two taxa of the Bithyniidae/Hydrobiidae and one Lithoglyphinae (*Bithynia tentaculata*, *Hydrobia sp.* and *Lithoglyphus naticoides*, respectively, see Table 5). In addition to one taxon sampled in this study (a previously unknown species of *Erhaia* from Guangxi, China), sequence data for two Amnicolidae were also taken from the GenBank. A variety of outgroup taxa was made available to the analysis in order to help detect any long branch attraction (LBA), which may affect the estimate of phylogeny, through the commonly applied method of taxon addition/removal and varying outgroup [123,124].

### Initial handling of data and selection of partitioning scheme and substitution models

Tajima’s test (based on the total number of mutations) and *Fu and Li’s test* for neutrality was performed using DNAsp (v. 5.10.01) [125]. The data were tested for substitution saturation using plots of the numbers of transitions (Ts) and transversions (Tv) against the F81 genetic distance [126]. The indications of these plots were further evaluated using the entropy-based test [84] as found in the DAMBE (v. 4.5.20) software package [127], which provides a statistical test for saturation. For all phylogenetic reconstructions (except for those using POY, which employed dynamic homology) indels were coded using Simple Indel Coding [128].

The incongruence length-difference (ILD) test [129] as implemented in PAUP* (v. 4.0b10; [130]) with 5000 replicates, was used to test for homogeneity between the *cox*1 and *rrn*L data partitions; the test was applied to informative sites only [131]. A suitable substitution model was selected for each data partition using an hierarchical test of alternative models as implemented in jModelTest (v. 2.1.2) [132,133], with default settings. Models were compared by AIC_C_. The starting parameters of the chosen models were then “optimized” using a Maximum Likelihood (ML) method with the Brent Powell algorithm in the phylogenetics software suite P4 (v. 0.90) [134]. Molecular evolution of indels was modeled by a F81 model, which is a monotone model fitting the similarly monotone substitution of a binary character evolving along a phylogeny [135]; however, in the analyses using Beast (v. 1.7.5) [136], a stochastic Dollo model [137] was also evaluated. It was considered that the Stochastic Dollo might provide a more realistic modelling of the indel process for our data if each indel is a unique, independent, event in the phylogeny. The Stochastic Dollo model here assumes that an indel is gained and then differentially lost in descendent lineages. No backward mutation is assumed. Each new indel is assumed independent of any pre-existing indel. The model has two rate parameters, an indel may arise at Poisson rate λ and is lost at constant rate μ [138]. The model was implemented both with and without correction for ascertainment bias (which may occur because the invariant sites have been excluded and are thus unobservable). The correction involves re-scaling the finite-time transition probabilities [137].

### Phylogenetic methods: parameters and model priors

Phylogenetic estimation used three different approaches; this strategy was adopted to look for resilience of the hypothesis to changes in method and associated assumptions. The rationale was that any clade that was represented in phylogenies found by all methods (and well supported) would be a reliable reconstruction of phylogenetic history for these taxa. In addition, the strategy increases the chance that artefacts peculiar to a specific class of methods will become apparent. For example, parsimony methods are perhaps more susceptible to the effects of long-branch attraction (LBA) than probabilistic methods [123,139] (although ML methods can also show LBA problems). Similarly, Bayesian methods have been known to erroneously converge on Long-Tree (LT) solutions in cases where partitioned data are described by complex models with many parameters close to nonidentifiability [140,141].

#### Likelihood based methods

A standard maximum likelihood method was applied using RAxML (v. 7.3.2) [142]. RAxML was chosen for this purpose because of its ability to handle partitioned data, whilst at the same time perform rapid bootstrapping and make use of parallel computing resources. RAxML also speeds ML search times using “Lazy Subtree Rearrangements” for branch swapping, and thus allows more replicates/multiple runs within the same time frame. RAxML also uses a simulated annealing approach, which incorporates a cooling schedule and allows “backward steps” during the hill-climbing process.

Bayesian phylogenetic analyses were implemented separately through two different packages, which were MrBayes (v. 3.2.1) [143] and Beast (v. 1.7.5) [136]. Bayesian approach (BA) was included in the analyses because these are not only faster in terms of computing time (for analyses with an equivalent level of confidence), but are also statistically superior to a method based on unintegrated likelihoods [144]. For example, such methods do not assume approximate normality or large sample sizes as would general ML methods [145]. BAs differ from direct ML methods in that the former consider the posterior probability of the model (with parameters) and tree after observing the data. The posterior probability of a hypothesis is proportional to the product of its prior probability and the probability of observing the dataset given the hypothesis (i.e., the likelihood of the hypothesis). Consequently, unlike direct ML, a BA allows incorporation of prior information about the phylogenetic process into the analysis. The analyses with Beast differed from those with MrBayes in that, with Beast, a molecular clock was applied to the tree and specified calibration point (date) priors were used in addition to substitution model parameter priors. Beast was also used to simultaneously estimate the divergence times for phylogenetic events relevant to phylogeographical hypotheses. The Bayesian approach implemented in Beast is currently considered superior to other approaches (e.g., non-parametric methods such as NPRS [81] or penalized likelihood methods [146], particularly for divergences with a low time depth, because it can allow for uncertainty in dates assigned to calibration points and does not require untested assumptions about the pattern of clock rate variation among lineages [147]. The greatest benefit of using a Bayesian method for dating is that the specification of prior distributions can be used to ensure that the analysis realistically incorporates the uncertainty associated with the calibration points used [148]. In the present study the indels were modelled by the TKF91 model (the default in Beast), which was found to perform better than the Stochastic Dollo. The settings particular to each phylogenetic method, and methods used in their selection, are described in detail in in Additional file 5 (Detailed description of methods). Details of priors used for dating in the Beast analyses are given below in the “Hypotheses testing” section.

#### Parsimony method using POY

POY (v. 5.0.0) [149] (parallelized build) was used to implement a Maximum Parsimony approach. The use of MP afforded an analysis free of the assumptions underlying ML methods, and the use of POY with its dynamic homology approach (where characters (transformation series) are inferred during the process of phylogenetic reconstruction) frees the analysis of any effects particular to the alignment inferred by Sequencher [150]. The choice of weighting schemes and run settings used followed published procedures [151].

### Hypotheses testing

#### Topology based method

The posterior probability of a particular phylogeographical hypothesis was tested by determining the proportion of trees with this topology in the posterior probability distribution (output from a tree search using MrBayes). This was done by writing a constraint tree describing the hypothesis of interest and reading this into PAUP*. Initially this was attempted using the trees in Figure 3, which represent the *a priori* hypotheses for a Tibet origin and a Hunan origin for the Triculinae; however, both of these trees had a posterior probability of zero because several of the taxa sampled had unexpected affinities. Consequently, it was necessary to modify both the *a priori* trees, in the same way, to allow for the unexpected positions of these taxa. The necessary modifications were, as follows:

i) All non-Pomatiopsidae (including *Jullienia rolfbrandti*) were restricted to an unresolved (polytomic) clade at the root of the tree (after the outgroup)

ii) *Tricula xiaolongmenensis* at root of the pomatiopsine clade

iii) *Tricula hortensis* at the root of all Triculinae, except for the West China Triculinae clade

iv) *Gammatricula sp.* and then *(G. fujianensis, J. jinghongensis)* at the root of all Sundaland Triculinae.

#### Shimodaira-hasegawa (SH) test

SH-tests [152] were performed as implemented in PAUP* with RELL optimization and 1000000 bootstrap replicates. The test compares between and among topologies from constrained and unconstrained searches; the set of 14 “best” trees from a MP analysis using PAUP* and the ML tree from an unconstrained PAUP* search were used as the set of trees for this comparison. The trees from Figure 3 (unedited) were used as the constraints for the two hypotheses under test. Although these two trees were written *a priori*, the use of the Kishino-Hasegawa test was not considered appropriate because the authors could not rule out the influence of earlier studies (some of which involved a sub-set of the present data) on the design of the trees. PAUP* was run with a CP_123_ and *rrn*L partitioning scheme, site specific rates, and the TPM1uf + I + G model with parameters as chosen by jModelTest for all sites combined. Gaps were treated as missing data.

#### Molecular dating method with beast

All Beast runs were performed with up to four date priors as calibration data. In reflection of the lack of certainty surrounding fossil dates, for example, divergence of taxa often pre-dates their first appearance in the fossil record [153], fossils maybe incorrectly assigned to extant taxa [154], and strata in which fossils formed may be eroded and subsequently overlain (leading to incorrect dating [155,156]), a wide time interval was used for each prior. Fossil dates are also subject to revision as new “earliest” fossils are discovered [73]. Molecular clock based calibrations often use clock rates set by fossil calibrations or palaeogeographical events (as in this study) and are, therefore, no more reliable than those based directly on fossils. Indeed molecular calibrations may suffer from the additional complications of rate variation among lineages or over evolutionary time [72]. As detailed below, measures were taken in the present study to minimize these problems. In addition to specifying wide ranging date priors, the priors were allowed to vary over a normal distribution such that the prior range (in most cases) corresponded to a 95% confidence interval. The HPD traces for parameter estimates were examined following each run to ensure that the estimates were not being truncated or overly skewed by the prior. Consequently, the prior effectively acts as a guide for the analysis, with the final estimates representing the result of an optimal combination of substitution model parameters, data, phylogeny and priors (this is a strength of all Bayesian methods). Care was also taken to achieve independence of calibration prior and hypothesis being tested.

Beast was run with the priors Root and Core-Group (Deep divergence runs) or Root, Core-Group Malaya and Khorat (Recent divergence runs). The “Root” prior provides a probability distribution for the TMRCA of the ingroup and *Lithoglyphopsis* (the root). The fossil record suggests that the Lithoglyphidae diverged from the Bithyniidae and Amnicolidae (i.e., from other hydrobioids) in the Jurassic [44]. In addition, fossil putative Rissooidea are known from the Carboniferous [157,158] and fossil hydrobioids are widespread by the upper Jurassic [159]. Consequently, the root of the phylogeny was dated as upper Carboniferous to mid-Jurassic (305–169 Ma). This time interval is also consistent with estimates based on a mt DNA clock (calibrated using minimum divergence dates from the littorinid fossil record) [44]. The Core-Group prior refers to the TMRCA of all the taxa except *Lithoglyphopsis* and *Hydrobia*, and is effectively the divergence date for the proto-Pomatiopsidae and the Amnicolidae. Fossil Bithyniidae are known from the Purbeckian [160], and the assimineid-pomatiopsid-truncatellid radiation is also thought to be Purbeckian [85]. The Purbeckian is roughly equivalent to the Berriasian [161]. Consequently, the upper range for this divergence was taken to be upper Jurassic. Hydrobiid taxa are seen to be well diverged (diverse) by the Santonian-Campanian, consequently the mid-Cretaceous was taken as the latest date for this range (i.e., 161–83 Ma, see Table 2). The prior Malaya (for the divergence of *Robertsiella* from the Sundaland *Neotricula* clade) was obtained from the divergence time estimated by a mt DNA molecular clock for *Schistosoma malayensis* and *S. mekongi*[60]; this clock was in turn calibrated using DNA substitution rates from the *S. incognitum* and *S. mansoni* divergence [162], which was based on hypotheses unrelated to those of the present study. Nevertheless, a normal prior (5.0 ± 1.0 Ma) was applied to the TMRCA of the *S. sinensium* group in the initial paper [60]. The prior was based on a date for the second major Himalayan orogeny; however, this was referring to events isolating Central Asia from the Orient [163], i.e., the acceleration of Tian Shan uplift at 5 Ma [88]. The prior is therefore not directly related to Himalayan events hypothesized in the Tibet or Hunan hypotheses, which mostly concern uplift in Southwest China.

Considering the more recent priors, the Malaya prior is based on *Schistosoma* and so assumes correspondence between the divergence of parasites and snails. It is not necessary to assume co-evolution, but merely that both the snails and parasites responded in the same way to the same events (i.e., Pleistocene changes in river courses where the snails live and eustatics). Also, although host-switching is not uncommon in *Schistosoma*, this tends to occur at higher taxonomic levels (of the parasites). As *Robertsiella* is the only pomatiopsid lineage found in peninsular Malaysia, and the entire *S. sinensium*-group is restricted to Pomatiopsidae, it is not unreasonable to assume that the ancestor of *S. malayensis* arrived in Malaysia through dispersal in the ancestor of *Robertsiella.* The prior Khorat is based on volcanism on the eastern margin of the Khorat Basin and flow reversal of the Mul river (*c.a.* 0.8 Ma), which are likely to have had an impact on the divergence of *Neotricula aperta*[19]. The precise dates of the more recent geological events are likely to be revised and refined in future geological studies and, as with the Malaya prior, appropriately wide confidence intervals were applied to this prior.

The calibration priors are summarised in Table 2 and were applied as normally distributed calibration dates to guide the inference of other divergence dates by Beast. The SD of each prior was chosen so that the 95% CI spanned the range of dates (obtained from the literature) given in column two of Table 2. Beast was then set to estimate the divergence times or TMRCAs of key clades in order to help test phylogeographical hypotheses.

Figure 6 shows the phylogenetic tree estimated by Beast (from recent dating runs) and the locations of each date prior and TMRCA estimated. The TMRCAs for the more recent clades were estimated separately from those of deeper divergences because Beast is currently unable to allow the clock rates to vary with time along a lineage. It is likely that clock rates will vary over long time scales, and attempts to estimate TMRCAs for divergences believed to have occurred 400–20 Ma simultaneously with those for divergences on the 15–0.5 Ma time scale, are unlikely to estimate the correct dates (at either scale) [147,164]. In general the molecular clock rate tends to be greater during evolutionary bursts or radiations (if these involve frequent population subdivision [165]) and along more recent lineages, than those in the deeper past, because in more recent lineages mutations may be present which, given sufficient time, will be removed by drift [166,167]. At the same time the effects of saturation accumulate in older divergences. Consequently, those TMRCAs expected to be Miocene or later were estimated separately from expectedly older divergences, and with the inclusion of more recent calibration date priors. Separating the estimates also leads to greater computational speed and replication when performing analyses on multiple machines/cpus.

A Jeffrey’s prior was placed on the clock rate for the indels (following the indications of test runs and BF comparisons). For the remaining partitions an informative prior was applied based on rates published in the literature for these loci. For the *cox*1 partitions a 1.75% clock rate was applied, with SD set to allow the rate to vary from 0 to 3.5% (at 95% confidence); the rate was allowed to vary to 35% for the third codon positions. For the *rrn*L data the rate prior was 1.097% (again with 95% CI covering 0-3%). A percent clock rate is equivalent to substitutions per site per year × 10^-8^. The rate priors were chosen with reference to the following sources. For *rrn*L, triculine snail divergences over the early Pleistocene [91], early Pliocene Hydrobiidae [168], and other divergences of Hydrobiidae [169]. The choice of rate for the *cox*1 data was guided by rates for Mio-Pliocene freshwater pulmonate snail divergence in Southeast Asia [151]. All runs were also performed with sampling from the prior only (no data) to ensure that the topology, clock rates, range of parameter estimates and tmrcas were not being directed by the priors over the data. Particular care was taken where a TMRCA was estimated for a divergence upon which a prior had also been specified (see initial paragraph of this sub-section).

### Availability of supporting data

DNA sequence data generated during this study are deposited in GenBank under accession numbers KC832687-KC832728 (http://www.ncbi.nih.gov/entrez/query.fcgi?db=Nucleotide&cmd=search&term=KC832687). All other data supporting the results of this article are included within the article and its additional files.

## Abbreviations

BA: Bayesian approach; BF: Bayes factor; CI: Confidence interval; HPD: Highest posterior density; ILD: Incongruence length-difference; LBA: Long branch attraction; LRT: Likelihood ratio test; Ma: Megaannum or million years; McMC: Markov chain Monte Carlo; ML: Maximum likelihood; MP: Maximum parsimony; rDNA: ribosomal DNA (ribosomal RNA gene cluster or operon); SD: Standard deviation; SE: Standard error; SEM: Scanning electron Microscopy; SH-test: Shimodaira-Hasegawa test; SIC: Simple indel coding; TMRCA: Time to the most recent common ancestor.

## Competing interests

The authors declare that they have no competing interests.

## Authors’ contributions

SWA Conceived and designed the study and analyses, and organized its undertaking. HGN, LLA and SWA performed the analyses. BJZ, HBH, HGN, LLA and SWA participated in the field sampling. BJZ, HBH and SWA were involved in identification of taxa. BJZ and HBH helped choose field sites and arrange access to sampling areas. SWA wrote the manuscript. All authors read and approved the final manuscript.

## Supplementary Material

Additional file 1: Figure S1 Phylogeny estimated by maximum likelihood. The maximum likelihood tree from a heuristic search (5 replicates) in PAUP* with the TPM1uf + I + G and site specific rates for the *cox*1 codon positions. Indels were not coded and all sites affected by indels were treated as missing data. The log likelihood of the tree was −11378.017. Click here for file

Additional file 2: Figure S2 A 50% majority-rule consensus tree for the BEAST analysis. This tree was estimated using the same data and analysis as for the maximum clade credibility tree in Figure 5. Posterior probabilities for each node are given. Click here for file

Additional file 3: Figure S3 POY strict majority-rule consensus tree. Tree estimated by maximum parsimony with dynamic homology using POY. The outgroup was set to be *Lithoglyphopsis naticoides* only. Click here for file

Additional file 4: Table S1 Taxa requiring revision and suggested affinities. Click here for file

Additional file 5 **Detailed description of methods.** Supplement to Section Phylogenetic methods: parameters and model priors. Details of phylogenetic methods: parameters, initial testing and model priors. Click here for file
