# Supplementary figures and images for: A phylogeny for the pomatiopsidae (Gastropoda: Rissooidea): a resource for taxonomic, parasitological and biodiversity studies
Source: BMC Evol Biol. 2014 Feb 18;14:29. doi: 10.1186/1471-2148-14-29 (PMC4016560; doi:10.1186/1471-2148-14-29)

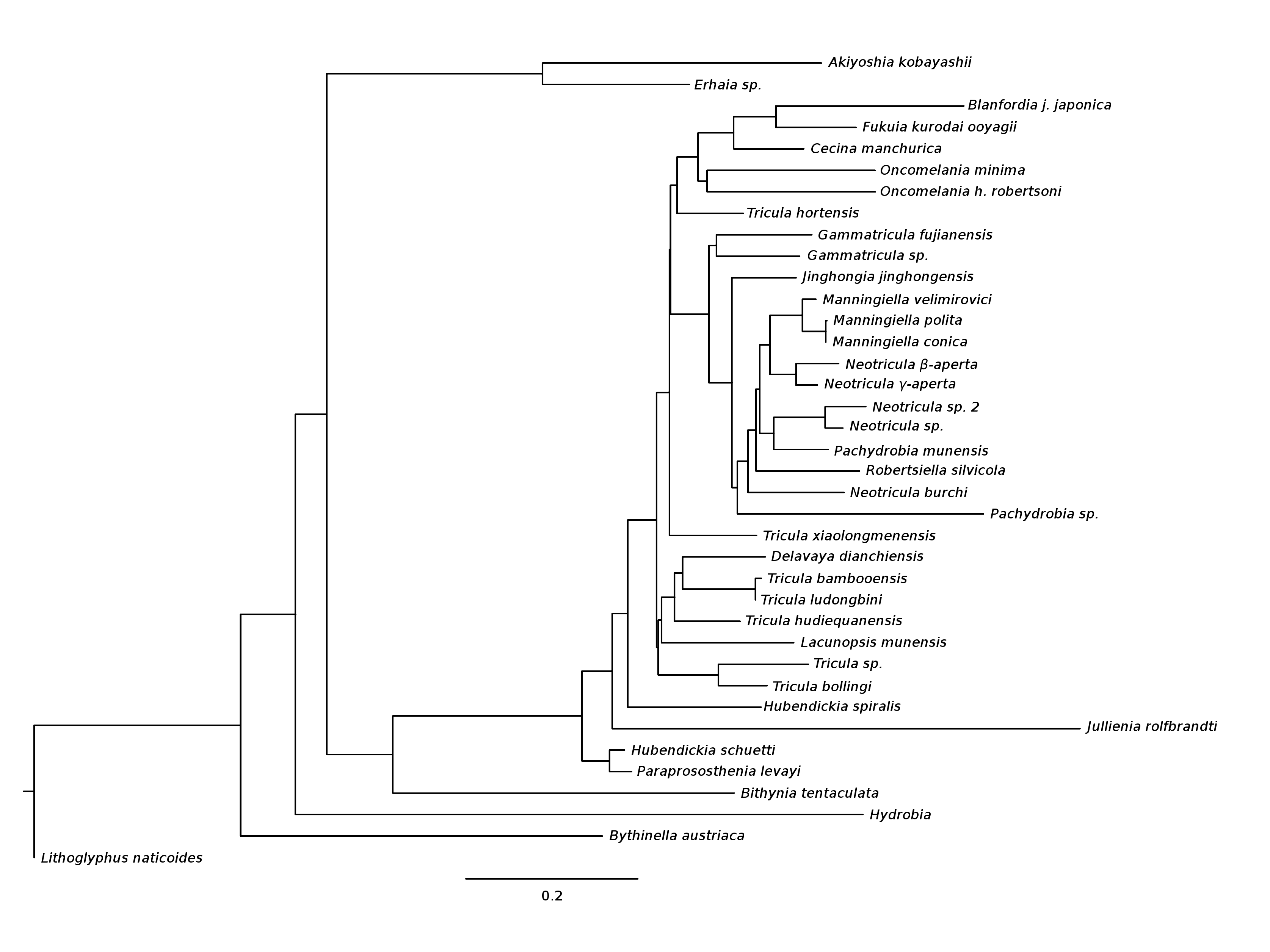

Supplement: Additional file 1: Figure S1 — Phylogeny estimated by maximum likelihood. The maximum likelihood tree from a heuristic search (5 replicates) in PAUP* with the TPM1uf + I + G and site specific rates for the cox1 codon positions. Indels were not coded and all sites affected by indels were treated as missing data. The log likelihood of the tree was −11378.017. [file 1471-2148-14-29-S1.png]

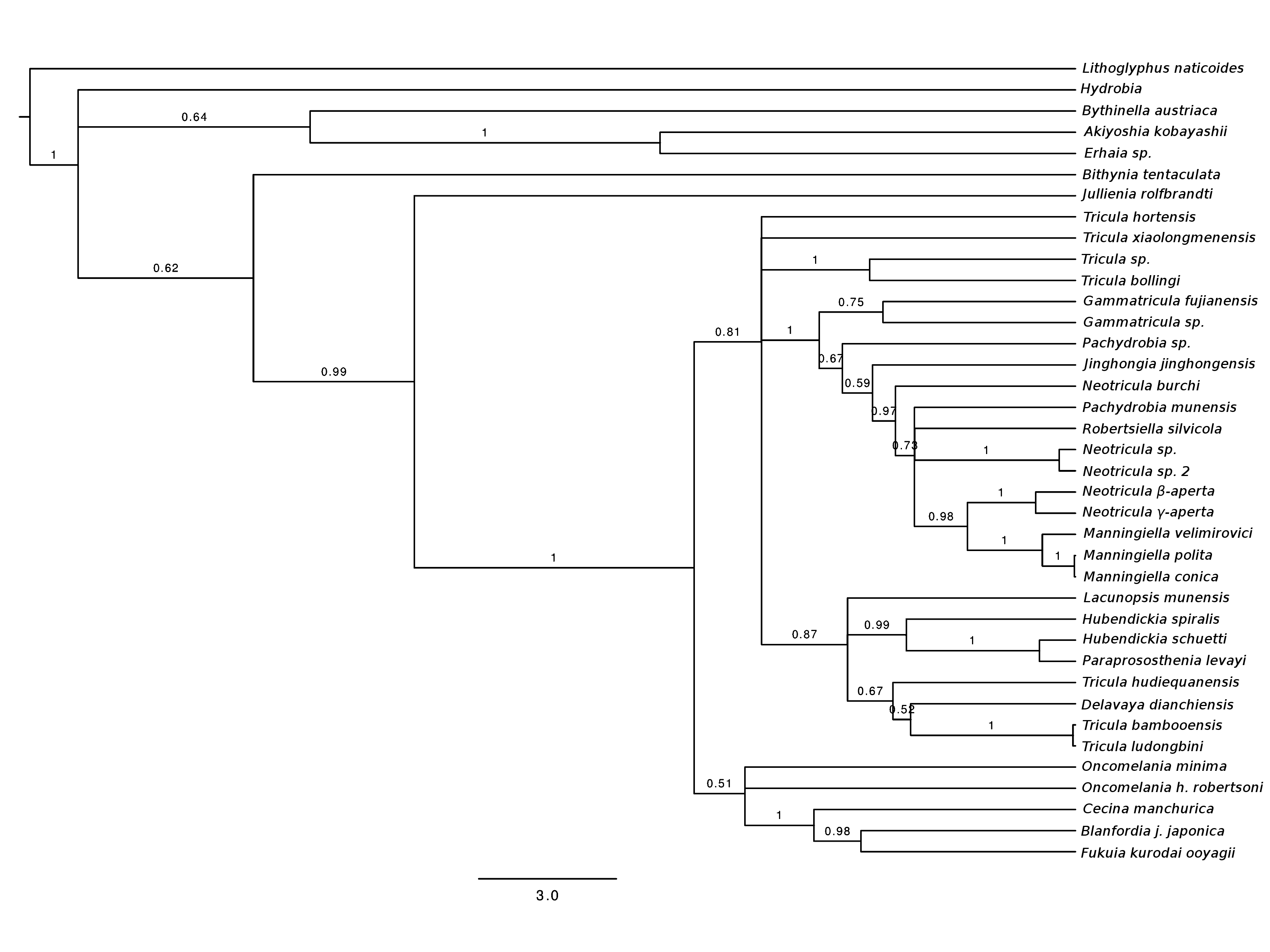

Supplement: Additional file 2: Figure S2 — A 50% majority-rule consensus tree for the BEAST analysis. This tree was estimated using the same data and analysis as for the maximum clade credibility tree in Figure 5. Posterior probabilities for each node are given. [file 1471-2148-14-29-S2.png]

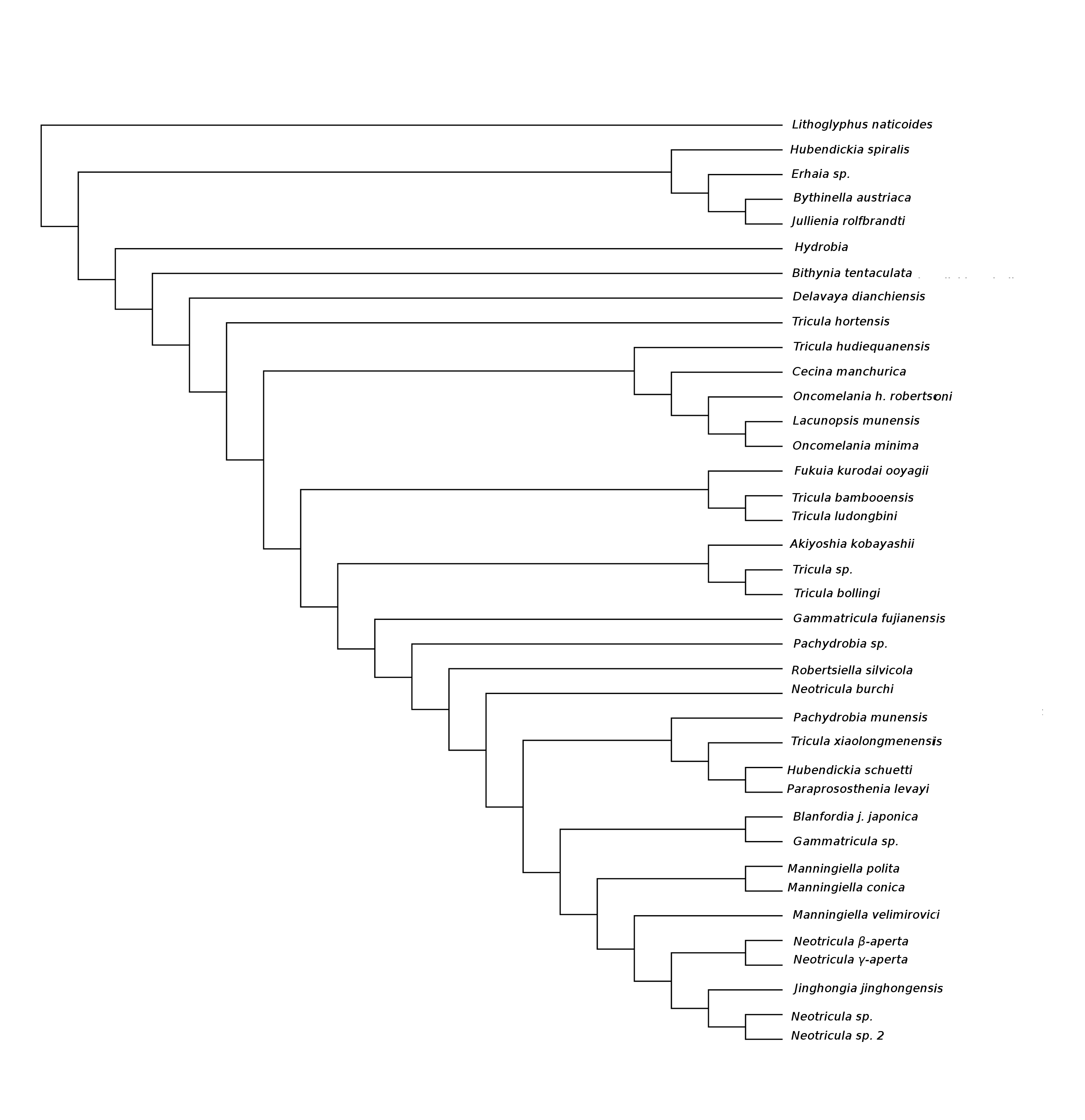

Supplement: Additional file 3: Figure S3 — POY strict majority-rule consensus tree. Tree estimated by maximum parsimony with dynamic homology using POY. The outgroup was set to be Lithoglyphopsis naticoides only. [file 1471-2148-14-29-S3.png]
