# Supplementary material for: A phylogeny for the pomatiopsidae (Gastropoda: Rissooidea): a resource for taxonomic, parasitological and biodiversity studies
Source: BMC Evol Biol. 2014 Feb 18;14:29. doi: 10.1186/1471-2148-14-29 (PMC4016560; doi:10.1186/1471-2148-14-29)
Supplement: Additional file 4: Table S1 — Taxa requiring revision and suggested affinities. [file 1471-2148-14-29-S4.doc]

**Table S1. Taxa requiring revision and suggested affinities.**

Species identified in the present study as in need of taxonomic revision are listed. The right-hand column provides a revised nomenclature; however, these suggestions are based on molecular differences and require confirmation by detailed morphological work, which is under way, and in some cases sampling of additional species in the same or related genera. Consequently, this Table is not a formal taxonomic revision. A taxon followed by a ? is a particularly tentative suggestion.

| *Tricula bambooensis* | *Delavaya bambooensis* |
| --- | --- |
| *Tricula ludongbini* | *Delavaya ludongbini* |
| *Tricula hudiequanensis* | *Delavaya hudiequanensis* ? (or new genus) |
| *Tricula sp.* (Mae Fang River) | *Tricula fontinalis* (new species) |
| *Hubendickia schuetti* | *Paraprososthenia schuetti* |
| *Gammatricula sp.* | *Gammatricula guangxiensis* |
| *Halewisia* (*Manningiella*) *conica* | *Neotricula conica* |
| *Halewisia* (*Manningiella*) *polita* | *Neotricula polita* |
| *Halewisia* (*Manningiella*) *velimirovici* | *Neotricula velimirovici* |
| *Neotricula burchi* | *Jinghongia burchi* ? (or new genus) |
