# Supplementary material for: A phylogeny for the pomatiopsidae (Gastropoda: Rissooidea): a resource for taxonomic, parasitological and biodiversity studies
Source: BMC Evol Biol. 2014 Feb 18;14:29. doi: 10.1186/1471-2148-14-29 (PMC4016560; doi:10.1186/1471-2148-14-29)
Supplement: Additional file 5 — Detailed description of methods. Supplement to Section Phylogenetic methods: parameters and model priors. Details of phylogenetic methods: parameters, initial testing and model priors. [file 1471-2148-14-29-S5.doc]

**Supplement to Section 5.4 (Phylogenetic methods: parameters and model priors)**

*Data partitioning and models*

The ILD test comparing the *cox*1 and *rrn*L loci with a common model (TPM1uf+I+G) was significant (P = 0.0002), indicating that the two partitions should be modelled separately (so that they may be analysed together). The starting models indicated for the ML analyses by jModelTest were as follows:

All characters, both loci: TPM1uf+I+G

cox1 first codon positions: TIM1ef+G

cox1 second codon positions: TIM1+I

cox1 third codon positions: TPM2uf+G

rrnL: TIM1+I+G;

however, the optimization of the parameters of these models in P4, and their use in Bayesian methods, allowed all rates to vary so that in effect the models were GTR (+ I and/or G, as above).

*Details of phylogenetic analyses with RaxML*

The ideal initial tree rearrangement value was determined for the data set as follows. RAxML was used to generate five different maximum parsimony starting trees and then run with these trees and RAxML's automatic determination setting turned on; the likelihood under the model and the topology of the “best tree” was noted for each of the five replicate runs. The procedure was then repeated for values of initial rearrangement setting ranging from 5 to 30, in increments of 5. The results were compared by Likelihood Ratio Tests (LRTs). No significant difference was found between the results of runs with the initial setting at 5 or at 30 (Δ 12.413, df 36, P=0.999). Consequently, the default initial rearrangement setting of 10 was used; therefore in the initial phase (Tree Bisection Reconnection (TBR)) of the search algorithm the pruned subtrees were inserted a distance of ≤ 10 nodes away from their original pruning point. Similarly, different values of the setting *e* were compared; this setting determines the likelihood difference down to which alternative topologies will be compared during the evaluation of the set of final trees, a larger *e* value would allow large sets of trees to be compared more rapidly. In the present study values of 0.01, 0.1, 0.5, 1.0 and 2.0 were tested. The searches were found to be quite robust to changes in *e*, with the LRT between e = 0.1 and 2.0 being non-significant (Δ 6.728, df 36, *P*≈1), consequently, the default value of 0.1 was again used. Finally, test runs were performed with a maximum parsimony starting tree or with a completely random tree, because the use of a random starting tree for data sets with only 100-200 taxa has been shown to sometimes yield topologies of distinct local likelihood maxima which better correspond to empirical expectations [1]. In the present study RAxML was run with a maximum parsimony starting tree because use of a random starting tree effected a slight fall in likelihood, although this was not significant (Δ 3.816, df 36, *P*≈1).

The analyses were performed with each codon of the cox1 sequence and the *rrn*L sequence partitioned separately (i.e., CP_123_ for the *cox*1 data). The indels partition was modelled with RAxML's model for binary substitution and using the Γ model of rate heterogeneity. RAxML was instructed to conduct a rapid Bootstrap analysis and search for the best-scoring ML tree in one single program run. The GTR model was used with the gamma correction for site rate heterogeneity and four discrete rate categories (alpha estimated for the Г-distribution by RaxML and unlinked across partitions). For the final runs RAxML was set to do 1000000 bootstrap replicates and then use every fifth tree found by bootstrapping as a starting tree for a series of 20000 full ML searches. The bootstrapping used RAxML's GTR+cat approximation (this is GTR with per site rate categories rather than GTR with the gamma correction). GTR+cat allows for integration of among site rate heterogeneity at a lower computational cost (approximately 4 times faster) and memory consumption (approximately 4 times lower). The technique provides a way to rapidly navigate into portions of the tree space where the trees score well under GTR+G [2]. The outgroup for the ML searches was *Lithoglyphus naticoides* only.

*Details of the use of the Bayesian phylogenetic method implemented in MrBayes*

In order to determine the most appropriate combination of options for analysis of the data, a series of short (1500000 generations) test runs was performed. The options tested were covarion on or off (and if on, switch rates linked or unlinked across the five partitions), the prior on the rates of the reversible rate matrix and the state frequency matrix as flat Dirichlet or as fixed to the optimized values from P4, the prior on the relative rates among partitions as flat Dirichlet or fixed to the relative rates from P4's optimization or as a Dirichlet prior based on P4's relative rates but with the slowest rate scaled to 1.0 and the other four rates scaled proportionally. Priors for Approximate Marginal Likelihoods (AMLs) (with respect to the prior) were obtained from the harmonic mean estimator of the marginal likelihoods for each model and Bayes Factors (BFs) (the ratio of the AMLs) [3] then used to compare the models' performance before and after a change in one of the settings. In addition to AMLs, the test runs were also examined for signs of failure to converge.

In order to help assess if runs had converged the average standard deviation of the split frequencies (ASDSF) was used to gauge the similarity of trees sampled by 2 independent runs and the Potential Scale Reduction Factor (PSRF) was used to compare the final mean parameter estimates (for the 2 runs), including the posterior probabilities of nodes [4, 5]. The McMC was considered complete if this ASDSF was <0.01 otherwise the run (and burnin) were considered insufficient. PSRF values close to 1.000 were also considered as indicative of possible convergence. The covarion option turns on or off the application of a modified covariotide model [6], which allows sites to independently switch between an ‘on’ and an ‘off’ state (a process regulated using the three default covarion matrices in MrBayes); this has the effect of allowing rate variation across the tree. The posterior distributions were examined using Tracer (v. 1.5) [7] and the run was accepted only if no distribution was seen to be cut off by its prior or show signs of failure to converge (rising likelihood). In addition, plots of log likelihood against generation number were used to ensure that the likelihood had reached a stable plateau suggestive of stationarity. Bayesian methods as implemented in MrBayes and similar applications are known to sometimes converge on an incorrect “Long Tree” (LT) solution where partitioned data are used with complex models. This can occur because the default starting tree used often has relatively long branch lengths for most data sets (this can draw the chain towards long-trees in the early stages of the run, such that convergence on the correct solution is never achieved). This problem may not be detected by either the ASDSF or PSRF [8]. To reduce the risk of this source of systematic error the following precautions were taken. Branch lengths of the final trees were compared with and without a user specified starting tree (the ML tree from RAxML was used for this). The relative rates for the partitions were examined because, as the chain is drawn to LT solutions the relative rate of the partition with fewest variable sites is often inflated (and alpha falls to accommodate this). In the present study the expectation was that third codon positions should have the highest rates and rrnL among the lowest - deviations from this would be considered symptomatic of an LT error.

All runs began with a random starting tree (except test runs as above) and estimation of parameters such as rate matrix, state frequencies, transition/transversion ratios, alpha of the Γ-distribution, and proportion of invariant sites, was unlinked across partitions. For the indel partition MrBayes was instructed to assume that only variable characters could be observed (a correction for ascertainment bias) and the F81 model was applied with only two character states. The prior on branch lengths was an exponential prior with a mean of 10 (default in MrBayes); however, values of 1.0 and 100 were also tested.

The results of the tests indicated the following settings/priors and these were used in the final runs. Covarion off, with switch rates unlinked (BF 40.34); mean on branch length prior 10 (BF, vs mean 1, is 28.43); relative rates among partitions Dirichlet (set to ratios of P4 optimized rates) (BF, vs Dirichlet flat, 11.48); rate prior on reversible rate matrix and state frequencies was fixed to P4 optimized values (BF, vs flat Dirichlet, 52.64). According to the table of Kass & Raftery [9], the BF values here are all very strong evidence against the hypotheses that the models compared were equivalent.

To improve the search of tree space, three final runs were executed with 28000000 generations each, a burnin of 80000 generations, and different random number seeds. The above number of generations was chosen as it exceeded the number used in previous studies, with similar sized data sets and loci [10] and had a convenient runtime (i.e., over Weekend). The sampling frequency was set so that each run produced 280001 samples of which 200001 samples were retained post-burnin. Four simultaneous Markov chains were run (one cold, three heated).

*Settings used in the Beast analyses*

Beast allows the user to specify a large number and variety of priors, preferences and models; the choice of these in the present study is detailed as follows. The clock model and the substitution model were unlinked across all five partitions; however, because the data were all mitochondrial loci, a single tree model was used for all partitions. Initially an uncorrelated relaxed log normal clock model was chosen for all partitions. An uncorrelated model was chosen because there was no reason to assume that rates in adjacent branches would be correlated – substitution rates may vary with generation time and life-cycle characteristics such as *r-*selection or *k-*selection, population stability and vagility; these are factors which can vary within clades of Triculinae such as within genera or even species (e.g., the strains of *N. aperta*). The log normal clock was chosen because it was expected that most rates would lie close to the mean for the overall phylogeny (rather than see many branch rates clustering at relatively low values). A pure Yule prior (effective to a prior on lineage divergence rate) was chosen for the tree prior, although one species of *Cecina* is known to have gone extinct; there is no evidence for major extinction of terminal lineages of Triculinae. A UPGMA starting tree was initially chosen because the analysis had many priors on the starting tree that had to be accommodated.

Initial test runs of only 71000000 generations were used to determine the ideal run settings. Comparisons were again made by means of BFs; however, the harmonic mean estimator was not used to approximate marginal likelihoods because this estimator is not only biased but also has a potentially very high variance. To overcome this problem the AMLs for all Beast runs were estimated using “Path Sampling”, an approach which, along side stepping-stone sampling, has been shown to outperform all other estimators [11].

The indels were modelled by either the TKF91 model (the default in Beast) or a stochastic Dollo model. The main drawbacks of the TKF91 are that it operates on single sites and employs a linear gap penalty [12, 13]. In cases where, for example, most deletions involve n sites (where n>1) the TKF91 model may not perform well. Further, sites that are lost in a deletion cannot easily be regained in a later insertion event (i.e., back mutation is less probable). Consequently, a Stochastic Dollo model was also tested for the modelling the evolution of the indels (see Methods, Section 5.3). Test runs indicated that the binary model performed significantly better than the Dollo model (BF 506, with correction for ascertainment bias) and so the simpler TKF91 model was used in the main analyses. Beast implements the SRD06 codon model for sequence data from protein coding genes; this combines the rates for the first and second codons, and appears to be a form of HKY_112_ + CP_112_ + Г_112_ model. The SRD06 was tested because it has been found to provide a better fit for protein coding nucleotide data [14]. Comparative runs of Beast with either the SRD06 model or with CP_123_ (with P4 optimized parameter values as priors) suggested that for these data CP_123_ performed significantly better (BF 3404). The code for the SRD06 model was generated using the SRD06 option in Beauti (v. 1.7.5) [14]. As a final test for the indels partition, runs were compared with or without correction for ascertainment bias and the TKF91 model; the findings were that performance was slightly better without the correction (BF 12).

The UPGMA starting tree or random starting tree was found to be often in conflict with one or more priors, and so the “best” tree from the MrBayes analyses was used as the starting tree in Beast. Those runs which did start with a random tree converged onto the same tree as found with the MrBayes tree, and so the user starting tree was incorporated into all further analyses. The MrBayes tree was scaled to be ultrametric with a height of 237 ± 34.7 (Ma), which is the prior on the age of the root (see “Root” Table 2) before use in Beast. The test runs also revealed that the confidence interval (CI) of the ucld.stdev included zero for the *cox*1 first and third codon positions and for the indels. Consequently, these partitions were run with a strict clock model in subsequent analyses.

In view of the findings of the test runs, the final Beast analyses were run with a Yule tree prior, the relaxed log-normal clock model on *cox*1 second codons and the *rrn*L partitions (and a strict clock on the other partitions), TKF91 model on the indels and settings as above (for MrBayes) on the other partitions, a user starting tree based on the tree found by MrBayes, and 180000000 generations chain length. In order to obtain the final estimate of phylogeny (tree) and estimates of time to the most recent common ancestor (tmrca) of clades of interest, Beast was run three times with identical settings but different random number seeds; the results of the three runs were then pooled (after removal of a 10% burnin) to give a net 486540000 generations sampled (540000000 generations pooled with a burnin of 53460000).

REFERENCES

1. Stamatakis A: *The RAxML 7.0.4 Manual*. 2006.

2. Stamatakis A: **Phylogenetic models of rate heterogeneity: a high performance computing perspective**. In *Proc. 20th Int. Conf. Parallel Distrib. Process.* Washington, DC, USA: IEEE Computer Society; 2006:253–253.

3. Newton MA, Raftery AE: **Approximate Bayesian inference by the weighted likelihood bootstrap**. *J. R. Stat. Soc. Lond.* 1994, **B56**:3–48.

4. Gelman A, Rubin DB: **Inference from Iterative Simulation Using Multiple Sequences**. *Stat. Sci.* 1992, **7**:457–472.

5. Ronquist F, Huelsenbeck JP: **MRBAYES 3: Bayesian phylogenetic inference under mixed models**. *Bioinformatics* 2003, **19**:1572–1574.

6. Huelsenbeck JP, Ronquist F, Nielsen R, Bollback JP: **Bayesian Inference of Phylogeny and Its Impact on Evolutionary Biology**. *Science* 2001, **294**:2310–2314.

7. Rambaut A, Drummond AJ: *Tracer*. Oxford: University of Oxford; 2004.

8. Brown JM, Hedtke SM, Lemmon AR, Lemmon EM: **When Trees Grow Too Long: Investigating the Causes of Highly Inaccurate Bayesian Branch-Length Estimates**. *Syst. Biol.* 2010, **59**:145–161.

9. Kass RE, Raftery AE: **Bayes Factors**. *J. Am. Stat. Assoc.* 1995, **90**:773–795.

10. Liu L, Mondal M, Idris M, Lokman H, Rajapakse PJ, Satrija F, Diaz J, Upatham ES, Attwood S: **The phylogeography of Indoplanorbis exustus (Gastropoda: Planorbidae) in Asia**. *Parasit. Vectors* 2010, **3**:57.

11. Baele G, Lemey P, Bedford T, Rambaut A, Suchard MA, Alekseyenko AV: **Improving the accuracy of demographic and molecular clock model comparison while accommodating phylogenetic uncertainty**. *Mol. Biol. Evol.* 2012:mss084.

12. Lunter G, Miklós I, Drummond A, Jensen JL, Hein J: **Bayesian Phylogenetic Inference under a Statistical Insertion-Deletion Model**. In *Algorithms Bioinforma.* edited by Benson G, Page RDM Berlin Heidelberg: Springer; 2003:228–244.

13. Miklós I, Lunter GA, Holmes I: **A “Long Indel” Model For Evolutionary Sequence Alignment**. *Mol. Biol. Evol.* 2004, **21**:529–540.

14. Drummond AJ, Suchard MA, Xie D, Rambaut A: **Bayesian Phylogenetics with BEAUti and the BEAST 1.7**. Mol. Biol. Evol. 2012, **29**:1969–1973.
